# Gene communities in co-expression networks across different tissues

**Published:** 2023-05-22

**Authors:** Madison Russell, Marie Saitou, Omer Gokcumen, Naoki Masuda

**Affiliations:** 1 Department of Mathematics, University at Buffalo; 2 Faculty of Biosciences, Norwegian University of Life Sciences; 3 Department of Biological Sciences, University at Buffalo; 4 Institute for Artificial Intelligence and Data Science, University at Buffalo

**Keywords:** multilayer networks, correlation matrix, community detection, modularity, gene modules

## Abstract

With the recent availability of tissue-specific gene expression data, e.g., provided by the GTEx Consortium, there is interest in comparing gene co-expression patterns across tissues. One promising approach to this problem is to use a multilayer network analysis framework and perform multilayer community detection. Communities in gene co-expression networks reveal communities of genes similarly expressed across individuals, potentially involved in related biological processes responding to specific environmental stimuli or sharing common regulatory variations. We construct a multilayer network in which each layer is a tissue-specific gene co-expression network. We develop methods for multilayer community detection with correlation matrix input and an appropriate null model. Our correlation matrix input method identifies groups of genes that are similarly co-expressed in multiple tissues (a community that spans multiple layers, which we call a generalist community) and some groups of genes that are co-expressed in just one tissue (a community that lies primarily within just one layer, which we call a specialist community). We further found gene co-expression communities where the genes physically cluster across the genome significantly more than expected by chance. This clustering hints at underlying regulatory elements determining similar expression patterns across individuals and cell types. The results indicate that our multilayer community detection method for correlation matrix input extracts biologically interesting communities of genes.

## 1 Introduction

In networks, communities, or modules, are broadly defined as groups of nodes with higher internal than external density of edges compared to a null model [1, 2]. There have been proposed numerous objective functions to be optimized and algorithms for community detection in networks. Because edges in networks represent a relationship between the nodes, it follows that these communities are groups of nodes that likely share common properties or play a similar role within the network. Many real-world networks naturally divide into communities, including biological networks, and studying communities is expected to help us better understand complex biological interactions [3–8].

Communities in gene networks are often called gene modules [4–6]. Methods to find functional gene modules are useful tools for discovering how the genes interact and coordinate to perform specific biological functions [9–12]. Furthermore, studying the relationships between gene modules may reveal a higher-order organization of the transcriptome [13, 14]. Biological analyses of gene modules can suggest genes that play a regulatory role in disease or other phenotypes, or identify novel therapeutic target genes for future intervention studies [15–18]. Additionally, one can study gene modules across evolutionary time to find biologically important groups of co-regulated genes because genes that must be co-expressed together will be under evolutionary pressure to maintain their coordinated expression [19, 20].

While there are various definitions of gene modules, or communities, in gene co-expression networks, gene modules are sets of genes that are similarly expressed across individuals and, therefore, potentially involved in related biological processes [16, 19, 21]. In such networks, the nodes represent genes, and a pair of nodes is directly connected with each other by an undirected edge if the two genes are co-expressed, i.e., if they show a similar expression pattern across samples [9, 15, 21–23]. Biologically, co-expressed genes may occur because transcription factors may have unique DNA binding sites located in promoter regions of distinct sets of genes [24, 25] , polymerase binding may cause synchronous transcription of several genes [26], physically closeby genes may cluster within similarly regulated topologically associated domains [27–29], or particular environmental factors may concurrently affect genes in a particular pathway [30–32], among other reasons [33]. Non-biological effects such as batch processing and RNA quality also contribute to gene co-expression [34, 35]. In general, one generally cannot distinguish between the biological and non-biological sources of co-expression from the expression data alone; thus, interpreting co-expression networks is challenging [33, 36]. However, gene co-expression network analysis may be able to clarify novel molecular mechanisms that are relevant to disease and facilitate identifying of potential targets for intervention studies [16, 33]. Crucially, gene coexpression and gene expression carry different information. For example, differential co-expression analysis identified the alpha synuclein variant (aSynL) in several Parkinson’s disease datasets. In contrast, differential expression analysis alone did not identify this variant since aSynL was highly differentially co-expressed but not highly differentially expressed [37]. Gene co-expression analyses can provide novel insights that are likely overlooked or undetected in traditional gene expression analyses [33].

Gene expression and co-expression may depend on regulatory elements in the genome, which are often specific to different cell types [17, 38–40]. The increased availability of tissue-specific gene expression data allows us to compare and contrast gene expression and co-expression and their communities across different tissues. A challenge for deciphering such data is integrating and distinguishing between communities found in various cell types, determining their biological relevance, and identifying regulatory elements maintaining these communities. For example, a simultaneous analysis of both generic multi-tissue co-expression (derived from aggregated gene expression data from multiple tissues) and tissue-specific co-expression resulted in a more efficient prediction of human disease genes than the use of generic multi-tissue co-expression alone [38]. It has also been found that modules conserved across different types of tissues are likely to have functions common to those tissues [39, 41]. In contrast, modules upregulated in a particular tissue are often involved in tissue-specific functions [39].

**Figure 1:**
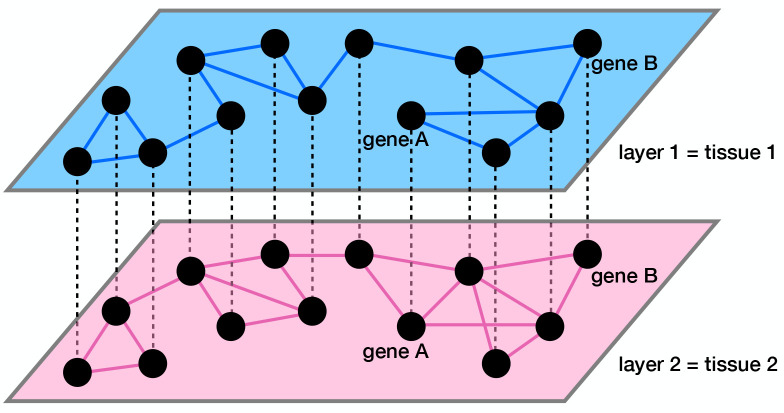
Schematic of a multilayer gene co-expression network. The intralayer edges, shown by the solid lines, represent co-expression. The interlayer edges, shown by the dashed lines, connect the same gene across layers.

One can regard a set of co-expression networks of genes constructed for multiple tissues as a multilayer network. As we schematically show in Figure 1, each layer of the multilayer network is a tissue-specific gene co-expression network. The edges within a layer (i.e., intralayer edges) represent tissue-specific co-expression. The edges between the layers (i.e., interlayer edges) connect the same gene across tissues. Multilayer network analysis, particularly multilayer community detection [42, 43], is becoming an increasingly popular tool in biological data analysis given that biological systems are often multi-dimensional and involve complex interactions [44–47]. Analyzing single-layer networks separately may be insufficient to reveal the patterns of these complex biological interactions [46]. For example, multilayer gene co-expression networks, in which each layer consists of a subset of gene pairs with a similar co-expression level, were constructed for comparing healthy and breast cancer co-expression patterns [48]. In the healthy multilayer co-expression network, the layers gradually attain hub nodes as one goes towards the top layer, whereas in the breast cancer multilayer network, the majority of layers contain no hub nodes and only a few top layers abruptly start to contain hub nodes [48]. Another application for multilayer approaches is to categorize diseases and drug targets. For instance, analyses of densely connected subgraphs that consistently appear in different layers have revealed disease modules (i.e., groups of diseases extracted from a four-layer disease similarity network in which a node is a disease and the four layers are constructed from protein-protein interaction (PPI), a symptom dataset, Gene Ontology, and Disease Ontology) [49] and drug-target modules (i.e., groups of genes extracted from a multilayer network in which each layer is a tissue-specific PPI network) [50]. Groups of diseases that have molecular and phenotypic similarities were discovered in an analysis of a bilayer network of human diseases consisting of a genotype-based and phenotype-based layers [51]. A multilayer network analysis in which each layer is a similarity matrix among 26 different populations for a given structural variant revealed evolutionarily adaptive structural variants [52]. Community detection on tissue-specific multilayer networks composed of a co-expression network, transcription factor co-targeting network, microRNA co-targeting network, and PPI network revealed candidate driver cancer genes [53].

As discussed above, the study of co-expression networks can lead to various biological insights [22, 33, 54]. However, there are some limitations to this approach. Edges of co-expression networks are correlational in nature. In general, creating unweighted or weighted networks from correlation data can be straightforward (e.g., thresholding on the edge weight and/or assuming no edges between negatively correlated node pairs). However, such straightforward methods are subject to various problems such as false positives [55, 56], arbitrariness in setting the parameter value such as the threshold on the edge weight [57, 58], and loss of information by subthreshold or negative correlation values [57, 59]. Methods to estimate sparse networks from correlation matrix data, such as graphical lasso [60–62] or estimation of sparse covariance matrices [63–65], mitigate some of these problems. In contrast to constructing sparse networks, in the present study, we explore the adaptation of network analysis methods to directly work on correlation matrix input. Such methods have been developed for community detection via modularity maximization [66–68] and clustering coefficients [69]. A key observation exploited in these studies is that one needs to use appropriate null models for correlation matrices, which are different from those for general networks. In particular, the standard null model for general networks called the configuration model, is not a correlation or covariance matrix in general [66]. In this study, we expand this line of approach to the case of multilayer correlation matrix data. In particular, we develop a method for community detection by combining multilayer modularity maximization and a configuration model of correlation matrices. We also construct statistical methods to calculate the significance of each detected community. We apply our methods to multilayer Pearson correlation matrices representing co-expression of genes in four tissues to compare communities of genes across different tissues. Code for running multilayer community detection with covariance matrix input is available at Github [70].

## 2 Methods

### 2.1 Data

The Genotype-Tissue Expression (GTEx) portal provides open-access tissue-specific gene expression data [71]. For the analyses in the present work, we use the gene transcripts per million (TPM) data from release V8 for four exocrine glands: pancreas, minor salivary gland, mammary gland, and skin (not sun exposed). In this pilot study, we limit our analysis to four tissues. We chose these tissues because they are all tissues that interact with the outside world and may have adaptively evolved to different environmental conditions. Specifically, the pancreas plays a vital role in the digestive system, secreting digestive enzymes [72]. The salivary gland is the main gatekeeper of our body and contributes to the oral proteome [73]. The mammary gland produces milk containing immunologic agents to nourish and protect young offspring [74]. The skin protects the body against pathogens, regulates body temperature, and has changed most drastically in human lineage [75, 76]. Consequently, we hypothesized that these tissues would retain a high level of variation in gene expression levels.

There are 328 samples from the pancreas, 163 samples from the minor salivary gland, 460 samples from the mammary gland, and 605 samples from the skin (not sun exposed) in this TPM dataset. Each sample contains gene expression data for 56, 200 different genes.

The number of genes is much larger than the number of samples for all tissues. Therefore, we focused on a subset of genes for our analysis around the same size as the number of samples in our data, as in [39,77]. To subset the genes, we identified the top 75 genes with the highest variance of TPM across all samples [22], separately for each tissue. We looked at the most variable genes because, again, our goal is to understand the underlying genetic and environmental bases of gene expression variation. In fact, most of the highly variably expressed genes are also highly expressed genes. To show this, for each tissue, we calculate the Jaccard index between the top 75 genes in terms of average TPM and the top 75 genes in terms of variance of TPM. The Jaccard index is defined as the size of the intersection of two finite sets A and B divided by the size of the union of A and B[78]. The range of the Jaccard index is 0 to 1, and a larger Jaccard index implies a greater overlap between the two sets of genes. We also examine the average rank of the top 75 genes in variance among all 56, 200 genes. We compute the rank in terms of the average TPM. Therefore, if the average rank is high (i.e., a low number), then the highly variable genes are also relatively highly expressed. We show in Table 1 the Jaccard index and the average rank of the top 75 genes for each tissue. The table indicates that the Jaccard index is at least 0.402 and the average rank is at most 167.8. These results suggest that the top 75 genes in terms of variance of TPM are overall highly expressed genes as well because we have calculated these indices for 75 genes in comparison to the 56, 200 genes.

The union of the top 75 genes in terms of the variance of TPM across the four tissues contains 203 genes. We analyze four-layer networks composed of these 203 genes.

### 2.2 Multilayer network construction

For each of the four tissues, we generate a 203 × 203 gene co-expression matrix in which the (i,j)-th entry is the Pearson correlation coefficient between the log-normalized TPM of gene i and the log-normalized TPM of gene j across all samples from that tissue. We take the logarithm of TPM before calculating the Pearson correlation coefficient to suppress the effect of outliers; TPM is extremely large for some samples. Let S denote the number of samples from tissue α. We denote by $x_{i,\alpha ,s}$ and $x_{j,\alpha ,s}$ the TPM value for gene i and j, respectively, for sample s∈{1,2,…,S} in tissue α. Then, we calculated the Pearson correlation coefficient between $log\left(x_{i,\alpha ,s}+1\right)$ and $log\left(x_{j,\alpha ,s}+1\right)$ across the S samples as the co-expression between gene i and gene j in tissue α. In other words, we calculate

(1) $$r_{\alpha }(i,j)=\frac{\sum_{s=1}^{S}\left[log\left(x_{i,\alpha ,s}+1\right)-m_{i,\alpha }\right]\left[log\left(x_{j,\alpha ,s}+1\right)-m_{j,\alpha }\right]}{\sqrt{\sum_{s=1}^{S}{\left[log\left(x_{i,\alpha ,s}+1\right)-m_{i,\alpha }\right]}^{2}\sum_{s=1}^{S}{\left[log\left(x_{j,\alpha ,s}+1\right)-m_{j,\alpha }\right]}^{2}}},$$

where

(2) $$m_{i,\alpha }=\frac{1}{S}\sum_{s=1}^{S}\log\left(x_{i,\alpha ,s}+1\right)$$

and

(3) $$m_{j,\alpha }=\frac{1}{S}\sum_{s=1}^{S}\log\left(x_{j,\alpha ,s}+1\right).$$

We took the logarithm of $x_{i,\alpha ,s}+1$ because, in this manner, $x_{i,\alpha ,s}=0$ is mapped to 0.

To compare the gene co-expression patterns across the different tissues, we view the four correlation matrices as a four-layer correlation matrix, or categorical layers of a multilayer gene co-expression network. Because the set of genes is the same in the four layers, we place an interlayer edge between the same gene in each pair of layers (i.e., tissues) as shown by the dashed lines in Figure 1. Therefore, our network is a multiplex network with diagonal and categorical interlayer couplings, where, by definition, the interlayer edges connect each gene with itself in each other layer [79, 80].

We denote the strength of the interlayer coupling that connects node i in layer α to node i in layer β as $\omega_{i\alpha \beta }$ [42]. One typically assumes that $\omega_{i\alpha \beta }$ takes binary values {0,ω}, where ω is a parameter indicating the absence (i.e., 0) or presence (i.e., ω) of interlayer edges [42]. However, how to set and interpret the ω value is not straightforward [81]. In this work, we use the empirical co-expression (i.e., Pearson correlation coefficient) of gene i between tissues α and β as $\omega_{i\alpha \beta }$. Specifically, $\omega_{i\alpha \beta }$ is equal to the right-hand side of Eq. (1) with $x_{j,\alpha ,s}$ and $m_{j,\alpha }$ being replaced by $x_{i,\beta ,s}$ and $m_{i,\beta }$, respectively, and with S being interpreted as the number of samples common to tissues α and β. Since the majority of studies on multilayer modularity maximization assume non-negative interlayer edge weights, if the obtained $\omega_{i\alpha \beta }$ is negative, we force $\omega_{i\alpha \beta }=0$. However, note that some studies do include negative interlayer edge weights [82].

### 2.3 Graphical lasso

For multivariate Gaussian distributions, a zero in the precision matrix (i.e., inverse covariance matrix) is equivalent to conditional independence of two variables, which one can relate to the absence of an edge in the network. Therefore, it is natural to use the non-zero entries of the estimated precision matrix to determine the edges [83]. However, when the number of variables is larger than the number of samples, the empirical covariance matrix is not full rank. In this case, the empirical covariance matrix is singular, meaning that its condition number is infinite, so estimating the precision matrix becomes difficult [62]. The graphical lasso addresses this problem by regularizing the maximum likelihood estimator with a lasso penalty enforcing sparsity [60–62].

Let $\vec{y}$ be a p-variate Gaussian random column vector, with distribution N(μ,C), where μ is the p-dimensional mean vector and C is the p×p covariance matrix. Given n independently drawn samples $\left\{\vec{y_{1}},\ldots ,\vec{y_{n}}\right\}$ of this random vector, the sample covariance matrix can be written as

(4) $$\hat{C}=\frac{1}{n-1}\sum_{k=1}^{n}\left(\vec{y_{k}}-\hat{\mu }\right){\left(\vec{y_{k}}-\hat{\mu }\right)}^{⊤},$$

where $\overset{ˆ}{\mu }=\frac{1}{n}\sum_{k=1}^{n}\vec{y_{k}}$, and ${\;}^{⊤}$ represents the transposition. Let the inverse covariance matrix be denoted as $C^{-1}=\Theta$. We consider a generalized $\ell_{1}$ regularization given by $\lambda \sum_{i=1}^{p}\sum_{j=1}^{i-1}\left|\Theta_{ij}\right|$, where λ is the penalizing parameter. Then, the problem is to maximize the lasso regularized log-likelihood to obtain the graphical lasso estimator, i.e.,

(5) $${\text{Θ}}^{\text{*}}=\text{arg}\;\underset{\text{Θ}≻0}{\text{min}}\left\{-\text{logdetΘ}+\text{tr}\left(\hat{C}\text{Θ}\right)+\lambda \sum_{i=1}^{p}\sum_{j=1}^{i-1}\left|{\text{Θ}}_{ij}\right|\right\},$$

where Θ≻0 signifies that Θ is a positive definite matrix [62].

Using the TPM data for each of the four tissues separately, we first calculated the 203 × 203 empirical covariance matrix. Then, we applied the GraphicalLassoCV function from a Python package sklearn to estimate a precision matrix, which is a sparsified co-expression network, for each tissue. This graphical lasso algorithm incorporates a cross-validated choice of the $\ell_{1}$ penalty. To simplify analysis, we regard the generated networks as unsigned and unweighted network. The results for the unsigned weighted networks are similar to those for the unsigned unweighted networks, as we will show.

### 2.4 Community detection in conventional multilayer networks

We are interested in detecting communities (also called modules and gene sets) in our multilayer networks to find sets of genes that are similarly expressed across individuals and therefore potentially involved in related biological processes. Some algorithms can detect communities that span between multiple layers as well as communities that lie within just one layer. We are interested in these different types of communities and their biological implications. A common method to find such communities in multilayer networks is to maximize an objective function called the multilayer modularity [42]. However, our multilayer gene networks are based on correlation. Therefore, we develop multilayer modularity for multilayer correlation matrices. In this section, we first explain multilayer modularity for usual multilayer networks as a primer to the multilayer modularity for correlation matrices and also as a tool for finding multilayer communities for our multilayer networks constructed with the graphical lasso. Then, we explain the derivation of the multilayer modularity for correlation matrix data and the statistical tests for testing the significance of individual communities.

#### 2.4.1 Multilayer modularity maximization

The modularity for single-layer undirected networks, which may be weighted, is given by [84, 85]

(6) $$Q=\frac{1}{2M}\sum_{i=1}^{N}\sum_{j=1}^{N}\left(A_{ij}-\gamma \frac{k_{i}k_{j}}{2M}\right)\delta \left(g_{i},g_{j}\right),$$

where N is the number of nodes in the given network; $A_{ij}$ is the (i,j)-th entry of the adjacency matrix and we assume $A_{ii}=0\;\forall i\in \{1,\ldots ,N\}$; $M=\frac{1}{2}\sum_{i=1}^{N}\sum_{j=1}^{N}A_{ij}$ is the number of edges in the case of unweighted networks and the total weight of all edges in the case of weighted networks; γ is the resolution parameter controlling the size of typical communities found by modularity maximization [86] ; large γ tends to lead to relatively many small communities; $k_{i}k_{j}/2M$ is equal to the probability that an edge exists, or alternatively the expected edge weight, between nodes i and j under the configuration model; $k_{i}=\sum_{j=1}^{N}A_{ij}$ is the (weighted) degree of node i; $g_{i}$ is the community to which node i belongs; $\delta \left(g_{i},g_{j}\right)=1$ if $g_{i}=g_{j}$ and $\delta \left(g_{i},g_{j}\right)=0$ otherwise.

To generalize the modularity to the case of multilayer networks, let L be the number of layers in the multilayer network. We let $A_{ij\alpha }$ be the (i,j)-th entry of the intralayer adjacency matrix, which may be weighted, in network layer α. We assume $A_{ii\alpha }=0\;\forall i\in \{1,\ldots ,N\}$ and ∀α∈{1,…,L}. We remind that $\omega_{i\alpha \beta }$ is the weight of the interlayer coupling between node i in layer α to node i itself in layer β. The multilayer modularity is given by [42]

(7) $$Q=\frac{1}{2\mu }\sum_{i=1}^{N}\sum_{j=1}^{N}\sum_{\alpha =1}^{L}\sum_{\beta =1}^{L}\left[\underset{\mathrm{intralayer}}{\underset{︸}{\left(A_{ij\alpha }-\gamma_{\alpha }\frac{k_{i\alpha }k_{j\alpha }}{2m_{\alpha }}\right)\delta_{\alpha \beta }}}+\underset{\mathrm{interlayer}}{\underset{︸}{\omega_{i\alpha \beta }\delta_{ij}}}\right]\delta \left(g_{i\alpha },g_{j\beta }\right),$$

where $k_{i\alpha }=\sum_{j=1}^{N}A_{ij\alpha }$ is the strength (i.e., weighted degree) of node i in layer α. We set $2\mu =\sum_{i=1}^{N}\sum_{\beta =1}^{L}\left(k_{i\alpha }+\left.\sum_{\beta =1}^{L}\omega_{i\alpha \beta }\right)\right.$, which is equal to the total edge weight. Let $\gamma_{\alpha }$ be the resolution parameter in layer α and $g_{i\alpha }$ be the community to which node i in layer α belongs. Equation (7) implies that communities that contain interlayer edges are rewarded with higher modularity values.

We will discuss the selection of $\gamma_{\alpha }$ in section 2.4.2. We use the Louvain algorithm for multilayer modularity maximization. Specifically, we use the iterated GenLouvain function, which repeatedly implements GenLouvain until convergence to an output partition (i.e., until the output partition does not change between two successive iterations) [87, 88].

The modularity function Q typically has many local maxima [89]. Reflecting this fact, most modularity maximization algorithms are stochastic and do not output a unique answer. A common approach to combine the results from multiple partitions of nodes is consensus clustering to obtain a consensus partition [90]. We use the consensus clustering algorithm described in [91] and implemented in the Python package netneuro-tools.

#### 2.4.2 Determining a resolution parameter value

For simplicity, we assume $\gamma_{\alpha }$ to be common for all layers and denote the common value by γ. We use the Convex Hull of Admissible Modularity Partitions (CHAMP) algorithm [92,93] to determine the γ value. The CHAMP algorithm takes a set of partitions generated by any community detection method as input and identifies the parameter regions in which each partition attains the largest modularity among all the partitions. The algorithm then obtains a pruned subset of admissible partitions and allows one to select parameter values corresponding to more robust community structures, which are large parameter regions in which the same partition maximizes the modularity.

Because we inform the interlayer coupling strength values by the empirical data, we only need to tune the γ value. Therefore, we input 15 partitions of the supra-adjacency matrix representing our multilayer network using evenly spaced γ values ranging from γ=1 to γ=4. Then, we employ the one-dimensional CHAMP to choose the optimal value of γ.

### 2.5 Community detection in multilayer correlation matrices

In this section, we expand modularity maximization for correlation matrices [66, 67] to the case of multilayer correlation matrices.

Let $\rho =\left(\rho_{ij}\right)$ be an N×N correlation matrix and ⟨ρ⟩ be a null model of the correlation matrix of the same size. The modularity for a single correlation matrix is given by

(8) $$Q=\frac{1}{C_{\text{norm}\;}}\sum_{i=1}^{N}\sum_{j=1}^{N}\left(\rho_{ij}-\langle \rho_{ij}\rangle \right)\delta \left(g_{i},g_{j}\right),$$

where $C_{\text{norm}}=\sum_{i=1}^{N}\sum_{j=1}^{N}\rho_{ij}$ is a normalization constant. One can use a modularity maximization algorithm to maximize Q given ⟨ρ⟩.

We generalize Eq. (8) to the case of a multilayer correlation matrix by writing down an equation in the same form as Eq. (7). We will use the term node to refer to a gene in a specific layer of the four-layer correlation matrix. Let $\rho_{ij\alpha }$ be the empirical Pearson correlation coefficient between nodes i and j in layer α, and let $\left\langle \rho_{ij\alpha }\right\rangle$ be the correlation between nodes i and j in layer α in the null model of the correlation matrix. Then, the modularity of a multilayer correlation matrix is

(9) $$Q=\frac{1}{C_{\text{norm}\;}}\sum_{i=1}^{N}\sum_{j=1}^{N}\sum_{\alpha =1}^{L}\sum_{\beta =1}^{L}\left[\left(\rho_{ij\alpha }-\gamma_{\alpha }\langle \rho_{ij\alpha }\rangle \right)\delta_{\alpha \beta }+\omega_{j\alpha \beta }\delta_{ij}\right]\delta \left(g_{i\alpha },g_{j\beta }\right),$$

where $C_{\text{norm}}=\sum_{j=1}^{N}\;\sum_{\beta =1}^{L}\left(\sum_{i=1}^{N}\rho_{ij\beta }+\sum_{\alpha =1}^{L}\omega_{j\alpha \beta }\right)$. Parameter $\gamma_{\alpha }$ represents the resolution in layer α [86], and we set their values using CHAMP, as we discussed in section 2.4.2. We remind that $\omega_{j\alpha \beta }$ is the empirical co-expression of gene j between tissues α and β. We double-count (i,j) and (j,i), with i≠j, in Eq. (9) following previous literature [66, 67].

We use a configuration model for correlation matrices [68] as the null model, while other null models are also possible, such as the H-Q-S algorithm [94] and those derived from random matrix theory [66]. The configuration model [68] generates the correlation matrix maximizing the entropy under the constraint that the strength (i.e., weighted degree) of each node of the input correlation matrix is conserved. To maximize Q given by Eq. (9), we feed the modularity matrix $B=\left(B_{ij}\right)$, where $B_{ij\alpha }=\left(\rho_{ij\alpha }-\gamma_{\alpha }\left\langle \rho_{ij\alpha }\right\rangle \right)\delta_{\alpha \beta }+\omega_{j\alpha \beta }\delta_{ij}$, to GenLouvain. Again, we use the iterated GenLouvain function [88] and a consensus clustering technique to obtain a final partition [91] but by inputting 200 partitions of the same network.

Prior studies developed methods to assess statistical significance of the detected communities in single-layer networks [9. 95, 97]. Here, we extend this approach to the case of multilayer correlation matrices and multilayer networks. We do this by comparing a detected community to the same set of nodes in a random graph (or null model) in terms of some quality measure. For each detected community and given quality measure, we calculated the Z score defined by

(10) $$z=\frac{x-\mu }{\sigma },$$

where x is the quality measure calculated for the empirical community, and μ and σ are the expected value and the standard deviation, respectively, of the same quality measure for the same community but under a null model. In the following text, we explain this method for multilayer correlation matrices, which we primarily use for our gene data analysis. We show the details of our methods for general multilayer networks in Supplementary Section S1.

We introduce a quality measure of a community that is analogous to the total weight of the intralayer edges within the community. Let W be the total weight of intralayer edges within the set of nodes S in a multilayer correlation matrix. In the remainder of this section, we use the covariance matrices instead of correlation matrices for analytical tractability. This assumption is not detrimental to the application of our methods to multilayer correlation matrix data because a correlation matrix is a covariance matrix in general. Let $C_{ij\alpha }^{\text{org}\;}$ be the (i,j)-th element of $C_{\alpha }^{\text{org}\;}$, an empirical covariance matrix for layer α. Then, we have

(11) $$W=\sum_{\alpha =1}^{L}\sum_{i=1\;(i,\alpha )\in S}^{N}\sum_{j=1\;(j,\alpha )\in S}^{i-1}C_{ij\alpha }^{\text{org}},$$

where (i,α) represents gene i in layer α, and the summation is over all node pairs ((i,α),(j,α)) in S. We exclude the diagonal elements, i.e., $C_{ii\alpha }^{org}$ in Eq. (11) because it is equal to 1 for correlation matrices.

Let $C_{\alpha }^{\text{con}\;}$ be a sample covariance matrix for layer α generated by the configuration model for correlation matrices [68]. Let $C_{ij\alpha }^{\text{con}\;}$ be the (i,j)-th element of $C_{\alpha }^{\text{con}\;}$. Using $E\left[C_{\alpha }^{\text{con}\;}\right]=C_{\alpha }$, where $C_{\alpha }$ is the covariance matrix for the estimated multivariate normal distribution for layer α [68], we obtain

(12) $$\text{E}\left[\sum_{\alpha =1}^{L}\sum_{i=1\;(i,\alpha )\in S}^{N}\sum_{j=1\;(j,\alpha )\in S}^{i-1}C_{ij\alpha }^{\text{con}}\right]=\sum_{\alpha =1}^{L}\sum_{i=1\;(i,\alpha )\in S}^{N}\sum_{j=1\;(j,\alpha )\in S}^{i-1}\text{E}\left[C_{ij\alpha }^{\text{con}}\right]=\sum_{\alpha =1}^{L}\sum_{i=1\;(i,\alpha )\in S}^{N}\sum_{j=1\;(j,\alpha )\in S}^{i-1}C_{ij\alpha }.$$

We obtain

(13) $$\mathrm{Var}\left[\sum_{\alpha =1}^{L}\sum_{i=1\;(i,\alpha )\in S}^{N}\sum_{j=1\;(j,\alpha )\in S}^{i-1}C_{ij\alpha }^{\text{con}\;}\right]=\frac{1}{L}\left[\sum_{\alpha =1}^{L}\sum_{i=1\;(i,\alpha )\in S}^{N}\sum_{j=1\;(j,\alpha )\in S}^{i-1}\sum_{k=1\;(k,\alpha )\in S}^{N}\sum_{r=1\;(r,\alpha )\in S}^{k-1}\left(C_{ik\alpha }C_{jr\alpha }+C_{ir\alpha }C_{jk\alpha }\right)\right].$$

We show the derivation of Eq. (13) in Supplementary Section S2. Note that

(14) $$\mathrm{Var}\left[\sum_{\alpha =1}^{L}\sum_{i=1\;(i,\alpha )\in S}^{N}\sum_{j=1\;(j,\alpha )\in S}^{i-1}C_{ij\alpha }^{\text{con}\;}\right]\propto \frac{1}{L},$$

which is consistent with the central limit theorem.

### 2.6 Specialist and generalist communities

The communities in multilayer correlation matrices and multilayer networks determined by the maximization of multilayer modularity may span multiple layers. We refer to a community containing genes belonging to various layers, i.e., tissues, as a generalist community. We refer to a community that contains genes in mostly just one tissue as a specialist community. We will give their precise definitions in the following text. These different types of communities occur due to the similarity or difference between gene co-expression patterns across different tissues. In particular, some pairs of genes show co-expression across individuals in only specific tissues and others in multiple tissues.

We define a measure called the specialist fraction to quantify how specialized any multilayer community is as follows. For a given community, we first find the number of genes unique to each tissue α, i.e., the genes i for which node (i,α) belongs to the community and node (i,β) does not for any β≠α. Second, we determine the specialist tissue of the community as the tissue that has the largest number of unique genes. The specialist fraction is the number of genes unique to the specialist tissue divided by the total number of nodes in the community. If the community lies within one layer, the specialist fraction is equal to 1. A large value of the specialist fraction suggests that the community is a specialist community. Genes unique to a specialist community may have functions specific to the tissue. In contrast, if all genes belong to at least two tissues, the specialist fraction is equal to 0. If many genes belong to different tissues in the community, the specialist fraction is low, suggesting that the community is relatively a generalist community. Genes in a generalist community may have functions expressed across various tissues.

### 2.7 Gene set enrichment analysis

To explore the biological processes associated with the set of genes constituting a detected community, we carried out a gene set enrichment analysis. It is a method for detecting statistically significant enriched biological processes, pathways, regulatory motifs, protein complexes, and disease phenotypes in the given gene set. We used g:Profiler for this purpose [98] and restrict our analysis to the Gene Ontology biological process (GO:BP) and Human phenotype ontology (HP) results. We use a Benjamini-Hochberg FDR significance threshold [99] of 0.05.

### 2.8 Localization of genes on chromosomes

We investigated whether the genes in a community detected by our method are physically clustered across the genome. To this end, we first asked whether a group of genes are more frequently located on the same chromosome than a control. Consider a group of genes, denoted by c. Let n be the number of genes in group c. We define the fraction of pairs of genes on the same chromosome as

(15) $$x_{c}=\frac{\;\text{number}\;\text{of}\;\text{pairs}\;\text{of}\;\text{genes}\;\text{in}\;\text{group}\;c\;\text{on}\;\text{the}\;\text{same}\;\text{chromosome}\;}{n(n-1)/2}.$$

The denominator of $x_{c}$ is equal to the number of pairs of genes in group c and gives the normalization. For the control, we uniformly randomly shuffle the association between the N=203 genes that we initially selected for our analysis and the chromosome to which each of the N genes belongs. After this random shuffling, the n genes are randomly distributed on various chromosomes as the N=203 genes are distributed on those chromosomes. Then, we calculate $x_{c}^{\text{rand}\;}$ according to this random distribution of the n genes using Eq. (15). We repeat this randomization 100 times and calculate the average and standard deviation of $x_{c}^{\text{rand}\;}$, and then the Z score. If the Z score is significantly positive, then we say that the group of genes c has more pairs of genes on the same chromosome than the control.

Second, we tested whether the genes in c are located closer to each other on the chromosome than a control, given the number of genes in c on each chromosome. To this end, we define the physical distance measured in base pairs between gene i and gene j on the same chromosome, d(i,j), as follows. Without loss of generality, assume that the end position of gene i is less than the start position of gene j. Then, we set

(16) d(i,j)=(startpositionofgenej)−(endpositionofgenei).

Furthermore, we define the average distance between genes in group c as

(17) $$d_{c}=\frac{\sum_{i,j}\text{in}\;\text{group}\;c\;\text{on}\;\text{the}\;\text{same}\;\text{chromosome}\;d(i,j)}{\;\text{number}\;\text{of}\;\text{pairs}\;\text{of}\;\text{genes}\;\text{in}\;\text{group}\;c\;\text{on}\;\text{the}\;\text{same}\;\text{chromosome}\;}.$$

Denote by $n_{k}$ the number of genes in group c that are on chromosome k. Note that denominator in Eq. (17) is equal to $\sum_{k}n_{k}\left(n_{k}-1\right)/2$. For the control, for each k, we choose $n_{k}$ genes uniformly at random out of all genes on chromosome k from the N genes. We carry out this procedure for all chromosomes k on which there are at least two genes in group c (i.e., $n_{k}\geq 2$). Then, we calculate $d_{c}$ for this random distribution of genes, which we refer to as $d_{c}^{\text{rand}\;}$. We repeat this randomization 100 times and calculate the average and standard deviation of $d_{c}^{\text{rand}\;}$, and then the Z score. If the Z score is significantly negative, then we say that the genes in group c are localized on the chromosomes.

Third, we tested whether the genes in c are located closer to each other than a control on a given chromosome. We define the average distance between genes in group c on chromosome k as

(18) $${\tilde{d}}_{c,k}=\frac{\sum_{i,j\;\text{in}\;\text{group}\;c\;\text{on}\;\text{chromosome}\;k}d(i,j)}{n_{k}\left(n_{k}-1\right)/2}.$$

For the control, we choose $n_{k}$ genes uniformly at random out of all genes that are among the N genes and on chromosome k. Then, we calculate ${\tilde{d}}_{c,k}$ for this random distribution of genes, which we refer to as ${\tilde{d}}_{c,k}^{\text{rand}\;}$. We repeat this randomization 100 times calculate the average and standard deviation of ${\tilde{d}}_{c,k}^{\text{rand}\;}$, and then the Z score. We carry out this procedure for each chromosome k on which there are at least two genes in group c (i.e., $n_{k}\geq 2$). We apply the Bonferroni correction [100] separately to each c to determine which communities have a significantly smaller average distance between pairs of genes on a specific chromosome than the control.

## 3 Results

### 3.1 Communities in the multilayer correlation matrix

We compare the gene communities obtained from the multilayer correlation matrix and those obtained from multilayer gene networks constructed using graphical lasso.

**Figure 2:**
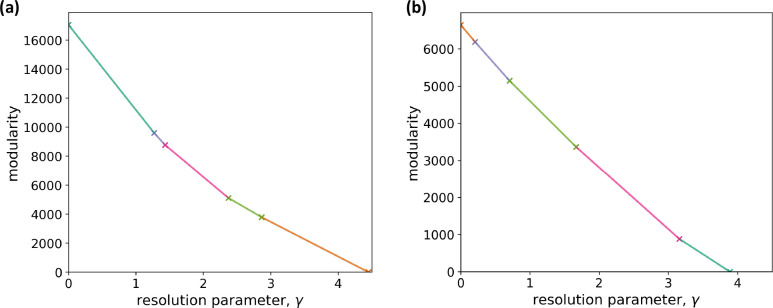
Determination of the resolution parameter value by CHAMP. (a) Multilayer correlation matrix. (b) Multilayer network obtained by graphical lasso. The convex hull of the lines in the (γ,Q) plane, each of which corresponds to a node partitioning, is a piecewise linear curve with the transition values indicated by a cross and change in the line color. Each line segment corresponds to the optimal node partitioning in the corresponding range of γ.

We run iterated GenLouvain [88] on the multilayer correlation matrix to approximately maximize the multilayer modularity at each value of the resolution parameter, γ, which we assume to be common for all layers. We then use the CHAMP algorithm to determine optimal values of γ [92, 93]. We show the results of the CHAMP in Figure 2(a). The figure indicates that robust ranges of γ, which are relatively wide ranges of γ in which the optimal partition is the same and correspond to relatively long straight line segments in the figure, are approximately 0<γ<1.2 or 2.9<γ<4.4. Therefore, we examine the node partitions with γ=1 and γ=3.

**Figure 3:**
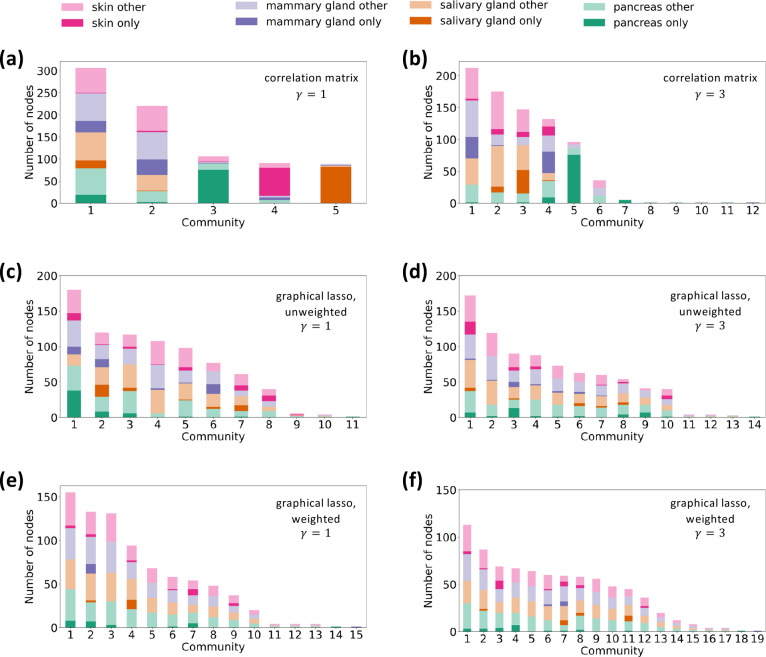
Composition of each community by layer, i.e., tissue. (a) Multilayer correlation matrix, γ=1. (b) Multilayer correlation matrix, γ=3. (c) Unweighted multilayer network obtained by graphical lasso, γ=1. (d) Unweighted multilayer network obtained by graphical lasso, γ=3. (e) Weighted multilayer network obtained by graphical lasso, γ=1. (f) Weighted multilayer network obtained by graphical lasso, γ=3. The darker shades represent the genes that only appear in one layer in the given community. The lighter shades indicate nodes corresponding to genes that appear in multiple layers in the community.

We show the composition of the resulting node partitions with γ=1 and γ=3 in Figures 3(a) and 3(b), respectively. As expected, the number of communities increases when γ increases. We show the Z score for the total intralayer weight within each community detected with γ=1 and γ=3 in Table 2 The communities labeled N/A contain no intralayer edges such that one cannot run the randomization, leading to the null Z score. These communities contain only one gene; communities 8, 9, 10, and 11 detected with γ=3 contain two nodes representing the same gene in two different tissues, and community 12 contains only one node. The table indicates that all the communities detected with γ=1 and all the communities containing at least two genes detected with γ=3 are statistically significant.

Both node partitions contain some communities that appear to be generalist communities and other communities that appear to be specialist communities. To quantify these findings, we show in Table 3 the specialist fraction for each community in the partition with γ=1. Communities 3, 4, and 5 have specialist fractions greater than 0.5, so we regard them as specialist communities. These three specialist communities represent three different tissues. In contrast, because communities 1 and 2 have specialist fractions substantially less than 0.5, we regard them as generalist communities. The same table also shows the specialist fraction for each significant community found with γ=3. Communities 5 and 7 have specialist fractions greater than 0.5. Both of them are pancreas specialist communities. We regard communities 1, 2, 3, 4, and 6, whose specialist fraction is substantially less than 0.5, as generalist communities.

### 3.2 Communities in the multilayer networks obtained by graphical lasso

For comparison purposes, we run the iterated GenLouvain on the multilayer networks that we constructed using graphical lasso. The results of CHAMP on the detected node partition of the unweighted network, shown in Figure 2(b), indicates that the optimal ranges of γ are approximately 0.7<γ<1.7 or 1.7<γ<3.2. Therefore, we use the same γ values as those for our multilayer correlation matrix, i.e., γ=1 and γ=3.

We show the composition of the resulting node partitions of the unweighted network obtained using graphical lasso with γ=1 and γ=3 in Figures 3(c) and 3(d), respectively. With γ=1, we find eleven communities, nine of which are significant. With γ=3, we find fourteen communities, ten of which are significant. See Supplementary Section S1 for the statistical results. We also show the composition of the node partitions of the weighted multilayer network obtained using graphical lasso with γ=1 and γ=3 in Figures 3(e) and 3(f), respectively.

Figures 3(c)–(f) suggest that these partitions apparently contain generalist communities only. Table 3 shows the specialist fraction for each significant community in the unweighted network and each community in the weighted network. Note that we have not evaluated the significance of the communities detected for the weighted multilayer network because the configuration model for weighted networks, which is necessary for constructing a significance test, is not a straightforward concept [101, 102]. For the unweighted network, with both γ=1 and γ=3, all the significant communities have specialist fractions at most 0.211. For the weighted network, with both γ=1 and γ=3, all the communities with more than one gene have specialist fractions at most 0.133. Therefore, we conclude that there are no specialist communities for either the unweighted or weighted network and with either γ=1 or γ=3. Because we are interested in comparing the biological implications of specialist communities versus generalist communities, in the following sections, we only analyze the communities detected for our multilayer correlation matrix.

### 3.3 Localization of genes on chromosomes

To investigate the possible localization of genes in the detected communities on the chromosomes, we first analyze whether the N=203 among the 56, 200 genes that we are analyzing in the GTEx data set are already localized in the genome. The Z score for a fraction of pairs of genes on the same chromosome is 6.735 $\left(p<10^{-6}\right)$, which suggests that the N=203 genes are distributed on different chromosomes in a highly biased manner relative to how all the 56, 200 genes are distributed. The Z score for the average distance between pairs of genes on the same chromosome is −6.059 $\left(p<10^{-6}\right)$. Therefore, the average distance between pairs of genes among the N=203 genes is significantly smaller than by chance. This result is expected given that highly expressed genes in glandular tissues cluster in specific loci [40]. We show the Z scores for the average distance between pairs of genes on each chromosome, analyzed separately, in Table 4 At a significance level of p=0.05, there is significant localization of genes on chromosomes 2 (p=0.0088; Bonferroni corrected; same for the following p values), 4 $\left(p<10^{-4}\right)$, 12 (p=0.0098), and 17 $\left(p<10^{-4}\right)$.

Next, we run the same localization analysis for each community in the multilayer correlation matrix detected with γ=1 and γ=3. For a generalist community, we only included the genes in the community that appear in at least three out of the four tissues in this analysis. This is because such genes may play functional roles, which the generalist community represents, across many types of tissues. With this restriction, each gene is present in at most one generalist community. Note that, without this restriction, a gene may appear in multiple generalist communities because the four nodes in the multilayer network representing the same gene may belong to different communities. We exclude this case for simplicity.

For each community, we show in Table 5 the Z score for the fraction of pairs of genes in the community that are on the same chromosome. With γ=1, communities 2 $\left(p<10^{-4}\right)$ and 3 $\left(p=4.05\cdot 10^{-4}\right)$ have significantly more genes among the N=203 genes on the same chromosome than by chance. The same table also shows the Z score for the average distance on the chromosome between pairs of genes in the same community for each community. We find that, with γ=1, community 2 has a significantly smaller average gene-to-gene distance than by chance (p=0.0336). With γ=3, communities 1 $\left(p<10^{-4}\right),$ 5 $\left(p<10^{-4}\right)$, and 6 $\left(p<10^{-4}\right)$ have significantly more pairs of genes on the same chromosome than by chance, and community 1 (p=0.0069) has a significantly smaller average gene-to-gene distance than by chance (see Table 5).

We then compute the Z score for the average distance between pairs of genes separately for each chromosome in addition to each community. We exclude the community-chromosome pairs that have less than three genes from this analysis. With both γ=1 and γ=3, no group of genes on a specific chromosome in a specific community is significantly clustered when we impose the Bonferroni correction over all the community-chromosome pairs (45 and 23 pairs with γ=1 and γ=3, respectively; see Supplementary Tables S3 and S4 for the Z scores). With the Bonferroni correction applied to each community separately, there are still no significant clusters in the partition with γ=1. However, with γ=3, we find that the genes in community 1 on chromosome 1 (p=0.0199) and those in community 5 on chromosome 11 (p=0.0371) are significantly clustered.

### 3.4 Functional analysis of selected communities

For the communities detected for our multilayer correlation matrix, we found clusters of physically localized genes within two communities with γ=3 but none with γ=1. Because we are interested in exploring biological implications of localized clusters of genes, we carry out further analysis on the node partition with γ=3 in this section.

First, we conducted an enrichment analysis of the communities identified with γ=3. We started by an enrichment analysis for the top 50 genes that have the highest expression out of the 203 genes in the network in each tissue. We find that, in all tissues, the top 50 highly expressed genes are enriched significantly in well-established housekeeping categories, such as oxidative phosphorylation and aerobic electron transport chain (FDR<0.05; see Supplementary Table S5). Echoing this finding, one of the modules that we identified (community 1) shows similar enrichment for mitochondrial function, such as aerobic electron transport chain $\left(p=1.05\cdot 10^{-10}\right)$ and oxidative phosphorylation $\left(p=5.90\cdot 10^{-11}\right)$ (see Supplementary Table S6). However, in the other six communities, our network approach identifies novel gene modules with functional enrichments in epidermis development (community 2, $p=1.90\cdot 10^{-24}$ ), keratinization (community 5, $p=1.95\cdot 10^{-19}$), positive regulation of respiratory burst (community 6, $p=5.36\cdot 10^{-8}$), and adaptive thermogenesis (community 7, $p=1.73\cdot 10^{-2}$). Furthermore, these modules are enriched with diseases relevant to the tissues examined, such as hyperkeratosis (community 2, $p=3.05\cdot 10^{-7}$) and recurrent pancreatitis (community 1, $p=1.78\cdot 10^{-19}$). Overall, our method provides additional biological insights than simple expression-level filtering.

**Figure 4:**
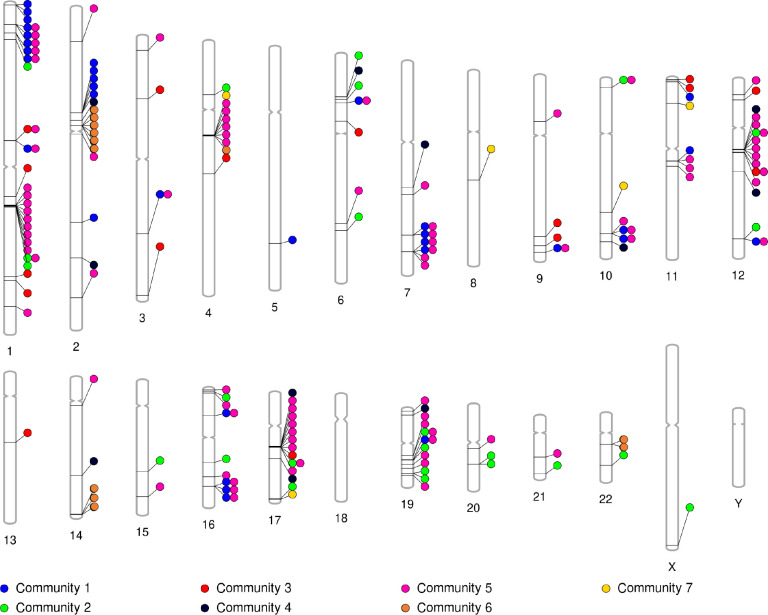
Location of genes on chromosomes, colored by community. There is a colored circle for each associated community pointing to each gene. Note that a gene can belong to more than one community, denoted by multiple colored circles next to each other horizontally pointing to the same gene. This figure allows us to visually see clusters of genes on specific chromosomes and their associated community.

Next we focused on investigating the physical clustering of co-expressed genes. We visualize in Figure 4 the location of the genes in the different communities on the chromosomes. As in the localization analysis presented in section 3.3, for a generalist community, we only show in Figure 4 the genes in the community that are present in at least three tissues. In Figure 4, a color of the circles represents a community. Note that a gene can belong to more than one community, denoted by multiple colored circles next to each other horizontally pointing to the same gene. It happens to be the case that a gene is associated with a maximum of two different communities, hence a maximum of two colored circles pointing to the same gene. Visually, Figure 4 suggests some tight clusters of genes, especially in community 5.

In section 3.3, we found significantly localized clusters of genes in community 1 on chromosome 1 and in community 5 on chromosome 11 in the partition with γ=3. It is somewhat surprising that only these two community-chromosome pair gene sets are significantly localized because there appear to be more localized clusters in Figure 4. A possible reason for this discrepancy is that, besides the genes in the community-chromosome pair of interest, there are so few other genes on the chromosome that the random shuffling of gene associations does not provide sufficient randomization. In this case, the empirical average distance between genes in the community-chromosome pair will not be statistically different from the average distance for the randomized data. Therefore, here we directly compared the average distance between pairs of genes on each community-chromosome pair, as defined by Eq. (18), to that for community 5 on chromosome 11. We decided to analyze community 5 because it is a pancreas specialist community while community 1 is a generalist community, as we discussed in regards to functional enrichment earlier in this section.

We denote the average distance between the pairs of genes among the three genes in community 5 on chromosome 11 by ${\tilde{d}}_{5,11}$, calculated using Eq. (18). We looked for any community-chromosome pair, containing all the genes in the selected community on the selected chromosome, with at least three genes whose average distance between genes is less than ${\tilde{d}}_{5,11}$. There are five such additional gene clusters: community 1 on chromosome M, which contains 15 genes, community 5 on chromosome 4, which contains 6 genes, community 5 on chromosome 17, which contains 9 genes, community 6 on chromosome 2, which contains 6 genes, and community 6 on chromosome 14, which contains 3 genes. Among all these community-chromosome pairs, we focused on the three gene clusters in community 5, including the gene cluster on chromosome 11. We opted to do so because community 5 is a pancreas specialist community, whereas communities 1 and 6 are generalist communities.

**Figure 5:**
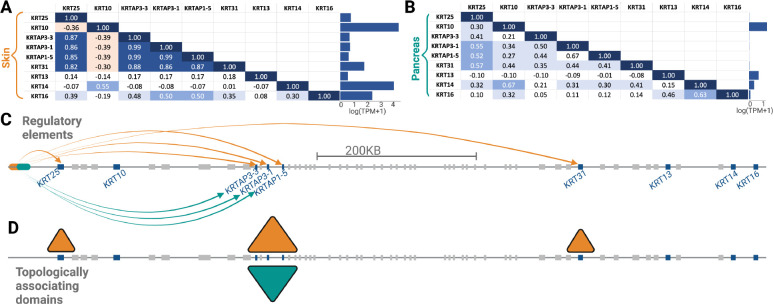
Expression and co-expression analysis of a cluster of genes in community 5 on chromosome 17. The co-expression matrices for these genes in (A) skin and in (B) pancreas are shown. The average expression for each gene in these tissues is shown in the bar graphs. The location of these genes on chromosome 17 is shown in (C), with arrows (colored according to the associated tissue) pointing from putative regulatory elements to highly co-expressed genes. (D) Topologically associating domains on the same region of the chromosome as that shown in C for skin and pancreas.

After initial investigation of the three gene clusters in community 5, i.e., one each on chromosome 4, 11, and 17, we further analyzed the one on chromosome 17, because keratin loci have been discussed in the context of human evolution [103, 104]. We show in Figure 5A and B the expression of each gene in this gene cluster in the skin and pancreas, respectively. We found that gene expression trends vary between the two tissues. Specifically, our method identified community 5 because of co-expression trends in the pancreas. However, in terms of the sheer expression level, the present gene cluster is expressed multiple folds higher in the skin than pancreas. Further, we found that the co-expression patterns for some gene pairs within this gene cluster are common between the skin and pancreas but differ for other gene pairs. The physical clustering of the genes that are co-expressed implicates genetic variation in shared gene regulatory factors as the main basis for co-expression. For example, a search of the GTEx eQTL database showed that the common single nucleotide polymorphism rs12450846 is significantly $\left(p<10^{-18}\right)$ associated with lower expression of KRT31 in the skin but higher expression of this gene in ovaries (p<0.005). Unfortunately, this analysis was not conducted in the pancreas. Regardless, this polymorphism and the haplotype linked to it regulate multiple other keratins and keratin-associated protein genes in this particular locus in a tissue and gene-specific manner according to the GTEx database. Thus, genetic variation that affects the efficacy of regulatory regions (Figure 5C) or the formation of topologically associated domains (Figure 5D) in a tissue-specific manner may underly the co-expression of the genes in community 5 on chromosome 17.

Another interesting community we identified is community 7. The genes in this community are located on different chromosomes and are enriched for response to temperature change (adaptive thermogenesis; see Supplementary Table S6). Because they exist on different chromosomes, it is unlikely that these genes share any common regulatory sequences or topologically associating domains. Instead, their co-expression may be due to environmental stimuli that are shared among the samples at the time of sampling (e.g., warm or cold environments). If true, the co-expression is due to a response to environmental stimuli that is controlled by specific regulatory sequences with broad effects across the genome, such as transcription factors. Thus, our network analysis may be useful for identifying gene clusters that respond to different environments.

## 4 Discussion

We developed a multilayer community detection method for Pearson correlation matrix data. We applied the proposed method to gene co-expression data from four tissues in humans to identify gene modules (i.e., communities). Some detected communities spanned multiple layers, which we refer to as generalist communities. Other communities lay mostly within one layer, specifically the pancreas layer, which we refer to as specialist communities. We then found that both generalist and specialist communities were localized on a smaller number of chromosomes than the expectation of random distribution of genes. Finally, as a case study, we closely looked into two gene clusters and suggested that the detected multilayer communities may imply gene regulatory factors shared across different tissues or environmental stimuli shared among samples.

Various mutually inclusive factors can explain co-expression of genes [16, 33, 34]. We explored two such factors in our case study. First, it is possible that the regulatory regions control the expression of multiple genes in certain tissues [24,26–29]. In this case, individuals who share genetic variations in these regulatory regions will have similar expression levels in these tissues where these regulators are active. If genetic variation underlies the co-expression of genes and the regulatory elements are cis (i.e., close physical proximity), we expect the co-expressed genes to cluster across the genome. We suggested that KRTAP3–3, KRTAP3–1, and KRTAP1–5 share regulatory elements in skin and pancreas. Indeed, several recent studies highlight topologically associating domains as potential sites underlying co-expression of multiple proximate genes [29,40]. Our approach integrated with chromatin accessibility (e.g., ATAC-seq) data is expected to facilitate identifying such loci where regulatory architecture may underlie the gene expression trends of multiple nearby genes in a tissue-specific manner. Second, it is possible that co-expressed genes have similar or complementing functions that respond to particular environmental conditions [30, 31]. For example, we suggested that the genes in community 7 detected in the multilayer correlation matrix with γ=3 may be involved in response to temperature change and co-expressed because samples were subjected to respective environmental conditions at the time of sampling. We argue that the response to environmental stimuli may underlie co-expression in these genes and thus indicate phenotypic plasticity for related traits [105], where an individual can respond to different environmental cues by adjusting the expression levels of multiple genes [106]. Our approach can provide a systematic framework to study phenotypic plasticity using animal models comparing different environmental stimuli (e.g., temperature, pathogenic pressure, diet, xenobiotic substances).

We employed multilayer modularity maximization. By design, modularity maximization consists in finding an optimal partition of nodes into non-overlapping communities, and therefore each node belongs to exactly one community. This feature is inherited to multilayer modularity maximization such that each node (i,α), where i represents a gene and α represents a layer, belongs to exactly one community. Multilayer modularity maximization has been used on biological networks to extract groups of proteins or genes that may be functionally related. For example, this technique was used on multilayer networks composed of transcription factor co-targeting, microRNA co-targeting, PPI, and gene co-expression networks as four layers for revealing candidate driver cancer genes [53] and on a multilayer network composed of pathways, co-expression, PPIs, and complexes networks for obtaining groups of disease-related proteins [107]. However, it is not straightforward to interpret the obtained multilayer communities as gene module because, within a single multilayer community, different genes appear in different sets of layers. For example, in a generalist community spanning all the four layers, some genes i may be present in all the layers, whereas other genes j may be present in only one layer. Then, although i and j belong to the same community and connected by group-level co-expression relationships, it may be difficult to argue that i and j share biological functions or environmental factors because how their co-expression depends on layers is different between genes i and j. One option to mitigate this problem is to focus on the resulting gene set in a given multilayer community and ignore the layer identity for simplicity [53, 107]. In contrast, we limited our analysis of generalist communities to the genes that appear in at least three out of the four layers in the community. In this manner, we argued that the genes in the generalist communities used in our localization and biological analyses may have functions common across different tissue types. For the two specialist communities that we analyzed in depth (with γ=3), we did not need to select genes because all genes were present in the pancreas and only a small fraction of genes were also present in a different tissue type.

The GTEx Consortium portal provides gene expression data from 30 types of tissues [71]. It is computationally straightforward to extend this analysis to more than four layers (i.e., tissues). Then, however, the results would quickly become much more complicated to interpret. With a number of layers much larger than four, it is likely that our method would no longer discover specialist communities. This is an important limitation of the present analysis. Developing methods more directly tailored to multilayer gene co-expression networks and correlation matrices with a larger number of tissues warrants future work. A suitable method should depend on biological questions. For example, enforcing pillar or semi-pillar communities such that all the genes belonging to the same multilayer community are present in the same set of layers [43,81] may facilitate biological interpretation of obtained results. Allowing overlapping of communities [108, 109] and genes not belonging to any community may be another choice. For example, overlapping community detection in single-layer networks has been shown to be better at identifying biologically relevant disease modules than non-overlapping community detection [108].

We only analyzed co-expression among N=203 out of the 56, 200 genes because it is difficult to reliably estimate covariance matrices when the number of samples is small [39, 77, 110–112]. Justifiable methods for analyzing co-expression matrices or networks of a larger number of genes are desirable. Such methods will enable us to reduce bias involved in choosing a small subset of genes to analyze. In contrast, a different approach is to formulate the estimation of large correlation networks from big data as a computational challenge and work on efficient algorithms and application to complex biological data [113]. Systematically investigating biological performance of network community detection as a function of the number of samples [112, 114, 115] will help us to better understand potentials and limitations of both single-layer and multi-layer community detection in gene and other related networks, which is left as future work.

## Supplementary Material

Supplement 1

## Figures and Tables

**Table 1:** Similarity between the highly variable genes and the highly expressed genes in each tissue. We calculate the Jaccard index between the top 75 genes in terms of average TPM and the top 75 genes in terms of variance of TPM. We calculate the average rank of the top 75 genes in variance, where the rank is in terms of average TPM.

Tissue	Jaccard index	Average rank

pancreas	0.685	52.81
salivary gland	0.531	64.31
mammary gland	0.402	133.5
skin	0.442	167.8

**Table 2:** Z scores for the total intralayer weight within each community detected in the multilayer correlation matrix. Comm. denotes community.

γ=1		γ=3	

Comm.	Z score	Comm.	Z score

1	24.432	1	68.526
2	73.282	2	55.267
3	62.569	3	72.008
4	14.972	4	19.071
5	65.318	5	124.080
		6	40.288
		7	14.699
		8	N/A
		9	N/A
		10	N/A
		11	N/A
		12	N/A

**Table 3:** Specialist fraction and the corresponding tissue for each community detected in the multilayer correlation matrix and for each community detected in the multilayer networks obtained by graphical lasso. Comm. denotes community and No. denotes “number of”.

Multilayer correlation matrix									

γ=1					γ=3				

Comm.	No. genes	No. specialist genes	Specialist fraction	Specialist tissue	Comm.	No. genes	No. specialist genes	Specialist fraction	Specialist tissue

1	153	26	0.085	mammary gland	1	96	34	0.160	mammary gland
2	104	35	0.159	mammary gland	2	84	9	0.051	salivary gland
3	92	76	0.717	pancreas	3	87	37	0.252	salivary gland
4	80	63	0.692	skin	4	88	34	0.258	mammary gland
5	86	82	0.921	salivary gland	5	86	76	0.792	pancreas
					6	12	0	0.000	N/A
					7	5	5	1.000	pancreas

Unweighted multilayer network obtained by graphical lasso									

γ=1					γ=3				

Comm.	No. genes	No. specialist genes	Specialist fraction	Specialist tissue	Comm.	No. genes	No. specialist genes	Specialist fraction	Specialist tissue

1	102	38	0.211	pancreas	1	80	18	0.105	skin
2	62	17	0.142	salivary gland	2	37	2	0.017	pancreas
3	48	6	0.051	pancreas	3	47	13	0.144	pancreas
4	36	2	0.019	mammary gland	4	33	4	0.045	skin
5	35	5	0.051	skin	5	21	2	0.027	mammary gland
6	35	14	0.182	mammary gland	6	24	4	0.063	salivary gland
7	32	8	0.131	salivary gland	7	18	2	0.033	salivary gland
8	17	8	0.200	skin	8	23	4	0.074	pancreas
9	3	1	0.167	skin	9	18	7	0.171	pancreas
					10	15	5	0.125	skin

Weighted multilayer network obtained by graphical lasso									

γ=1					γ=3				

Comm.	No. genes	No. specialist genes	Specialist fraction	Specialist tissue	Comm.	No. genes	No. specialist genes	Specialist fraction	Specialist tissue

1	51	8	0.052	pancreas	1	35	3	0.027	pancreas
2	54	11	0.083	mammary gland	2	30	3	0.034	pancreas
3	38	3	0.023	pancreas	3	31	9	0.130	skin
4	38	11	0.117	salivary gland	4	23	7	0.104	pancreas
5	17	0	0.000	N/A	5	16	0	0.000	N/A
6	16	1	0.017	pancreas	6	19	2	0.033	mammary gland
7	24	7	0.130	skin	7	32	5	0.085	salivary gland
8	12	0	0.000	N/A	8	23	3	0.052	salivary gland
9	12	3	0.081	skin	9	14	0	0.000	N/A
10	5	0	0.000	N/A	10	12	0	0.000	N/A
					11	19	6	0.133	salivary gland
					12	11	2	0.056	skin
					13	5	0	0.000	N/A
					14	3	0	0.000	N/A
					15	2	0	0.000	N/A

**Table 4:** Z score for the average distance between pairs of genes on each chromosome for the *N* = 203 genes. M stands for the mitochondrial chromosome. Chr denotes chromosome.

Chr	Z score	Chr	Z score

1	−1.881	14	0.085
2	−3.540	15	−0.404
3	1.293	16	0.599
4	−4.736	17	−4.842
5	−1.858	18	N/A
6	−0.422	19	−2.545
7	−0.873	20	−1.458
8	0.691	21	−0.317
9	0.276	22	−0.564
10	0.552	X	2.103
11	−1.112	Y	N/A
12	−3.512	M	−0.858
13	N/A		

**Table 5:** Analysis of localization of genes in each community detected in the multilayer correlation matrix. Note that $x_{c}$ is the normalized fraction of pairs of genes in the community on the same chromosome and that $d_{c}$ is the normalized distance between two genes in the community on the same chromosome. Comm. denotes community.

γ=1			γ=3		

Comm.	Z score for $x_{c}$	Z score for $d_{c}$	Comm.	Z score for $x_{c}$	Z score for $d_{c}$

1	1.520	−1.095	1	6.190	−3.251
2	7.094	−2.710	2	−0.388	0.652
3	3.940	−1.245	3	0.879	−0.405
4	0.160	0.179	4	−0.281	0.924
5	−0.102	−0.344	5	4.485	−1.175
			6	6.634	−2.255
			7	−0.845	N/A
