# Supplementary material for: Gene communities in co-expression networks across different tissues
Source: ArXiv. 2023 Dec 7:arXiv:2305.12963v2. Originally published 2023 May 22. Preprint. [Version 2] (PMC10246089)
Supplement: 1 — S1 Text. Fig A. Composition of each community by layer, i.e., tissue, for the multilayer correlation matrix originating from the 150 genes with the highest variance of TPM in each tissue, detected with our community detection method for multilayer correlation matrices with γ=3. Although there are 50 communities detected, we only show the communities with more than one gene in this figure. The darker shades indicate nodes corresponding to genes that only appear in one layer in the given community. The lighter shades indicate genes corresponding to genes that appear in multiple layers in the community. Fig B. Jaccard index between the set of tissue-specific hub genes and the set of genes in a community. Each row corresponds to the top 50 hub genes in each layer (i.e., tissue), where “panc” denotes pancreas, “sal” denotes salivary gland, “mamm” denotes mammary gland, and “skin” denotes skin (not sun exposed). Each column corresponds to a community identified with γ=3. Table A. Z scores for the number of intralayer edges within each community and for the conductance of each community detected in the unweighted multilayer network obtained by graphical lasso with γ=1 and γ=3. Comm. denotes community and no. denotes “number of”. Table B. Z scores for the average distance between pairs of genes on each chromosome and each significant community detected with γ=1. Comm. denotes community and Chr denotes chromosome. Table C. Z scores for the average distance between pairs of genes on each chromosome and each significant community detected with γ=3. Comm. denotes community and Chr denotes chromosome. Table D. Results of the gene set enrichment analysis for the top 50 highly expressed genes out of the 203 genes in the network in each tissue. Table E. Results of the gene set enrichment analysis for the communities of the multilayer correlation matrix with γ=3. Comm. denotes community. Table F. Results of the gene set enrichment analysis for the top 50 highly connected genes out o [file NIHPP2305.12963V2-supplement-1.pdf]

Supplementary Information for:

Gene communities in co-expression networks across different tissues

Madison Russell, Alber Aqil, Marie Saitou, Omer Gokcumen, Naoki Masuda

S1 Text

Text A. Analysis of an expanded multilayer correlation matrix

To validate our choice of the top 75 genes in each tissue in terms of the variance of TPM, we repeated the same analysis with the top 150 genes in each tissue in terms of the variance of TPM. The union of the top 150 genes across the four tissues contains 371 genes. We analyze a four-layer correlation matrix composed of these 371 genes. We run our community detection method for multilayer correlation matrices with $\gamma = 3$, which is the main value of γ used in the analysis in the main text. We show the partition of the 371-gene multilayer correlation matrix in Fig A.

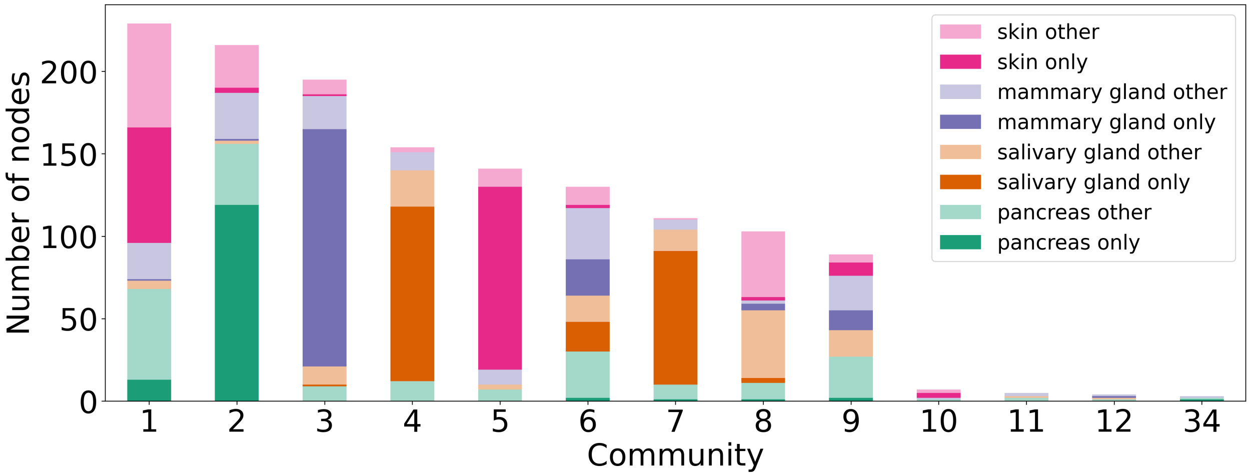

Fig A. Composition of each community by layer, i.e., tissue, for the multilayer correlation matrix originating from the 150 genes with the highest variance of TPM in each tissue, detected with our community detection method for multilayer correlation matrices with $\gamma = 3$. Although there are 50 communities detected, we only show the communities with more than one gene in this figure. The darker shades indicate nodes corresponding to genes that only appear in one layer in the given community. The lighter shades indicate genes corresponding to genes that appear in multiple layers in the community.

We find that the expanded multilayer correlation matrix with 371 genes produces a similar type of partition as the original multilayer correlation matrix with 203 genes, in the sense that there are some specialist communities and some generalist communities. We compare the larger of the pancreas specialist communities in the original correlation matrix (i.e., community 5 in the main text, shown in Fig 3(b)) to the pancreas specialist community in the expanded correlation matrix (i.e., community 2 in Fig A). Community 5 in the original correlation matrix contains 86 genes in the pancreas layer, and 88% of these genes also appear in community 2 in the pancreas layer of the expanded correlation matrix. Hence, there is significant overlap between the pancreas specialist communities of the two correlation matrices. This result supports robustness of our analysis with respect to the choice of the number of genes selected for our analysis.

Text B. Significance of communities detected in general multilayer networks

B.1 Null model

We assume undirected and unweighted networks. A common choice for a null model is the configuration model, in which the degree sequence $\{k_i\}_{i=1}^{N}$ is specified, and an edge is laid between nodes i and j with probability

$$p_{ij} = \frac{k_i k_j}{2M}, \tag{S1}$$

where k_i is the degree of the ith node in the original network. However, we avoid the configuration model for two reasons. First, by imposing the value of $k_1, \ldots, k_N$, the stochastic generation of edges sharing a node is not independent of each other. For example, if k_1 is small and we have generated edge $(1, 2)$ with probability p_{12}, then edge $(1, 3)$ is generated with a probability smaller than p_{13}. This type of correlation makes it difficult to analytically derive the quality measure of individual communities that requires the count the edges sharing a node. Second, large $k_i k_j$ values can yield $p_{ij} > 1$ [1]. One could set a structural cutoff degree $k_{\max}$ to be of the order of $\sqrt{N}$ to enforce the constraint $p_{ij} < 1$ [2]. However, it is often the case that the largest degree in an empirical network far exceeds this structural cutoff value [3, 4].

Instead of the configuration model, we use an exponential random graph model (ERGM) as the null model. Instead of the exact degree, we fix the expected degree of each ith node to k_i^*, the degree of the same node in the original network. Let Ω be an ensemble of networks with N nodes. Let $\vec{\theta} \equiv (\theta_1, \ldots, \theta_N)$ be the model parameters. The probability distribution of the adjacency matrix, $A = (A_{ij})$, that maximizes the Shannon entropy subject to the constraints

$$\sum_{A \in \Omega} P(A) k_i(A) = k_i^*, \tag{S2}$$

where $k_i(A)$ is the degree of the ith node in network A, and the normalization condition

$$\sum_{A \in \Omega} P(A) = 1 \tag{S3}$$

is

$$P(A|\vec{\theta}) = \prod_{i=1}^{N} \prod_{j=1}^{i-1} p_{ij}^{A_{ij}} (1 - p_{ij})^{(1 - A_{ij})}, \tag{S4}$$

where

$$p_{ij} = \frac{e^{-\theta_i - \theta_j}}{1 + e^{-\theta_i - \theta_j}} \tag{S5}$$

is the probability that nodes i and j are adjacent [5–7].

We infer the model parameters $\vec{\theta}$ by maximizing the associated log-likelihood function, i.e.,

$$\mathscr{L}(\vec{\theta}) \equiv \ln P(A^*|\vec{\theta}) = -\sum_{i=1}^{N} \theta_i k_i^* - \sum_{i=1}^{N} \sum_{j=1}^{i-1} \ln(1 + e^{-\theta_i - \theta_j}), \tag{S6}$$

where A^* is the adjacency matrix of the original network. One can derive Eq. (S6) from Eq. (S4).

We numerically determine θ_i's using a fixed point method implemented in the Python package NEMtropy [7]. We use NEMtropy to generate this so-called undirected binary configuration model (UBCM) [7]. We estimate the UBCM for each layer independently. Using the obtained multilayer UBCM as null model, we calculate the statistical significance of individual communities detected in the original multilayer network.

B.2 Number of intralayer edges within each community

We use two measures to assess the quality of individual communities. The first measure is the number of intralayer edges within each community, which is essentially the same as X used in the main text for correlation matrices. Previous studies used the number of edges within a community in single-layer networks [8, 9], and we extend this quality measure to multilayer networks.

Let S be a set of nodes in a multilayer network. Let X_α be the number of edges within S in layer α, and let X be the total number of intralayer edges within S, i.e.,

$$X = \sum_{\alpha=1}^{\mathcal{L}} X_\alpha. \tag{S7}$$

In the UBCM, the edges are independently laid. Therefore, X obeys the Poisson binomial distribution, which is the discrete probability distribution of a sum of independent Bernoulli trials that are not necessarily identically distributed [10].

Let $\theta_{i\alpha}$ be the UBCM parameter for node i in layer α. Using Eq. (S5) for each layer, we obtain the expectation of X as follows:

$$\mathrm{E}[X] = \sum_{\alpha=1}^{\mathcal{L}} \sum_{\substack{i=1 \\ (i,\alpha)\in S}}^{N} \sum_{\substack{j=1 \\ (j,\alpha)\in S}}^{i-1} \frac{e^{-\theta_{i\alpha}-\theta_{j\alpha}}}{1+e^{-\theta_{i\alpha}-\theta_{j\alpha}}}, \tag{S8}$$

where the summation is over all node pairs $(i,\alpha),(j,\alpha)$ in S. Note that we have excluded self-loops. The variance of X is equal to [10]

$$\mathrm{Var}[X] = \sum_{\alpha=1}^{\mathcal{L}} \sum_{\substack{i=1 \\ (i,\alpha)\in S}}^{N} \sum_{\substack{j=1 \\ (j,\alpha)\in S}}^{i-1} \left(1 - \frac{e^{-\theta_{i\alpha}-\theta_{j\alpha}}}{1+e^{-\theta_{i\alpha}-\theta_{j\alpha}}}\right) \frac{e^{-\theta_{i\alpha}-\theta_{j\alpha}}}{1+e^{-\theta_{i\alpha}-\theta_{j\alpha}}}. \tag{S9}$$

B.3 Conductance of each community

The second quality measure is the conductance of each community. Let $G(V,E)$ be an undirected single-layer network, and we consider a set of nodes $S \subseteq V$. Let

$$c_s = |\{(u,v) \in E : u \in S, v \notin S\}| \tag{S10}$$

be the number of edges on the boundary of S and

$$m_s = |\{(u,v) \in E : u \in S, v \in S\}| \tag{S11}$$

be the number of edges within S. Then, the conductance of S is given by [9]

$$\varphi(S) = \frac{c_s}{2m_s + c_s}. \tag{S12}$$

The conductance measures the fraction of the number of half-edges emanating from nodes in S that are connected to a half-edge emanating from a node outside S. Therefore, the conductance is small for a good community [11].

To define the conductance of a set of nodes S in a multilayer network with $\mathcal{L}$ layers, let Y_α be the number of edges on the boundary of S in layer α. We define the conductance of S by

$$\varphi(S) = \frac{\sum_{\alpha=1}^{\mathcal{L}} Y_\alpha}{\sum_{\alpha=1}^{\mathcal{L}} (2X_\alpha + Y_\alpha)} = \frac{Y}{2X+Y}, \tag{S13}$$

where Y is the number of intralayer edges on the boundary of S. Because the UBCM independently lays edges for different node pairs, Y as well as X obeys a Poisson binomial distribution. It should also be noted that X and Y are independent because they are calculated based on disjoint sets of node pairs.

We denote the set of intralayer node pairs within community S by

$$E_{\max}^{\mathrm{within}} = \{(i,j,\alpha) : (i,\alpha) \in S, (j,\alpha) \in S, i<j\}. \tag{S14}$$

We note that the cardinality (i.e., number of elements) of $E_{\max}^{\mathrm{within}}$ is equal to

$$x_{\max} \equiv \sum_{\alpha=1}^{L} \frac{N'_{S\alpha}(N'_{S\alpha}-1)}{2}, \tag{S15}$$

where $N'_{S\alpha}$ is the number of nodes in S in layer α. Because X obeys the Poisson binomial distribution, the probability for X is

$$P_X(X = x) = \sum_{\overline{E} \subset E_{\max}^{\text{within}} \text{ s.t. } \left|\overline{E}\right| = x} \prod_{(i,j,\alpha) \in \overline{E}} p_{ij\alpha} \prod_{(i,j,\alpha) \in E_{\max}^{\text{within}} \setminus \overline{E}} (1 - p_{ij\alpha}), \tag{S16}$$

where

$$p_{ij\alpha} = \frac{e^{-\theta_{i\alpha} - \theta_{j\alpha}}}{1 + e^{-\theta_{i\alpha} - \theta_{j\alpha}}}, \tag{S17}$$

and x is the number of edges in S. Similarly, the probability for Y is

$$P_Y(Y = y) = \sum_{\overline{E} \subset E_{\max}^{\text{boundary}} \text{ s.t. } \left|\overline{E}\right| = y} \prod_{(i,j,\alpha) \in \overline{E}} p_{ij\alpha} \prod_{(i,j,\alpha) \in E_{\max}^{\text{boundary}} \setminus \overline{E}} (1 - p_{ij\alpha}), \tag{S18}$$

where

$$E_{\max}^{\text{boundary}} = \{(i, j, \alpha) : (i, \alpha) \in S, (j, \alpha) \notin S\}. \tag{S19}$$

Note that the cardinality of $E_{\max}^{\text{boundary}}$ is

$$y_{\max} = \sum_{\alpha=1}^{\mathcal{L}} N'_{S\alpha}(N - N'_{S\alpha}). \tag{S20}$$

Because X and Y are mutually independent, the expected value of the conductance of a set of nodes S in the multilayer network is given by

$$\mathrm{E}\left[\frac{Y}{2X + Y}\right] = \sum_{x=0}^{x_{\max}} \sum_{y=0}^{y_{\max}} \frac{y}{2x + y} P_X(X = x) P_Y(Y = y). \tag{S21}$$

The variance of the conductance of S is given by

$$\begin{aligned} \mathrm{Var}\left[\frac{Y}{2X + Y}\right] =& \sum_{x=0}^{x_{\max}} \sum_{y=0}^{y_{\max}} \left(\frac{y}{2x + y}\right)^2 P_X(X = x) P_Y(Y = y) \\ &- \left[\sum_{x=0}^{x_{\max}} \sum_{y=0}^{y_{\max}} \frac{y}{2x + y} P_X(X = x) P_Y(Y = y)\right]^2. \end{aligned} \tag{S22}$$

In Eqs. (S21) and (S22), we set $\frac{y}{2x+y} = 1$ for $(x, y) = (0, 0)$.

B.4 Results

We show in Table A the Z scores for the number of intralayer edges within each community and for the conductance of each community detected in the unweighted multilayer network obtained by graphical lasso with $\gamma = 1$ and $\gamma = 3$. For $\gamma = 1$, communities 10 and 11 are each composed of a single gene; for $\gamma = 3$, communities 11 through 14 are each composed of a single gene. We omitted these single-gene communities in Table A. Except these single-gene communities, for both $\gamma = 1$ and $\gamma = 3$, all other communities are statistically significant with a large positive Z score for the number of intralayer edges and a large negative Z score for the conductance.

Table A. Z scores for the number of intralayer edges within each community and for the conductance of each community detected in the unweighted multilayer network obtained by graphical lasso with $\gamma = 1$ and $\gamma = 3$. Comm. denotes community and no. denotes "number of".

$\gamma = 1$			$\gamma = 3$		
Comm.	Z score for no. intralayer edges	Z score for the conductance	Comm.	Z score for no. intralayer edges	Z score for the conductance
1	26.787	−35.697	1	25.702	−31.396
2	43.706	−49.785	2	50.807	−60.543
3	41.245	−49.208	3	39.556	−42.975
4	49.956	−60.620	4	32.401	−34.112
5	42.446	−45.851	5	49.755	−52.415
6	43.054	−49.280	6	48.585	−52.584
7	34.795	−37.814	7	43.897	−46.904
8	29.940	−31.740	8	37.580	−40.672
9	57.664	−62.551	9	36.172	−37.726
			10	30.597	−32.125

Text C. Derivation of the variance of the total intralayer weight for a community in a multilayer correlation matrix

To derive Eq. (11) in section 2.4, we start with

$$\begin{aligned}
&\mathrm{Var}\left[\sum_{\alpha=1}^{\mathcal{L}} \sum_{\substack{i=1\\(i,\alpha)\in S}}^{N} \sum_{\substack{j=1\\(j,\alpha)\in S}}^{i-1} C^{\mathrm{con}}_{ij\alpha}\right]\\
=&\mathrm{E}\left[\left(\sum_{\alpha=1}^{\mathcal{L}} \sum_{\substack{i=1\\(i,\alpha)\in S}}^{N} \sum_{\substack{j=1\\(j,\alpha)\in S}}^{i-1} C^{\mathrm{con}}_{ij\alpha}\right)^2\right] - \left(\mathrm{E}\left[\sum_{\alpha=1}^{\mathcal{L}} \sum_{\substack{i=1\\(i,\alpha)\in S}}^{N} \sum_{\substack{j=1\\(j,\alpha)\in S}}^{i-1} C^{\mathrm{con}}_{ij\alpha}\right]\right)^2\\
=&\mathrm{E}\left[\sum_{\alpha=1}^{\mathcal{L}} \sum_{\substack{i=1\\(i,\alpha)\in S}}^{N} \sum_{\substack{j=1\\(j,\alpha)\in S}}^{i-1} \sum_{\beta=1}^{\mathcal{L}} \sum_{\substack{k=1\\(k,\beta)\in S}}^{N} \sum_{\substack{r=1\\(r,\beta)\in S}}^{k-1} C^{\mathrm{con}}_{ij\alpha} C^{\mathrm{con}}_{kr\beta}\right] -\\
&\sum_{\alpha=1}^{\mathcal{L}} \sum_{\substack{i=1\\(i,\alpha)\in S}}^{N} \sum_{\substack{j=1\\(j,\alpha)\in S}}^{i-1} \sum_{\beta=1}^{\mathcal{L}} \sum_{\substack{k=1\\(k,\beta)\in S}}^{N} \sum_{\substack{r=1\\(r,\beta)\in S}}^{k-1} C_{ij\alpha} C_{kr\beta}\\
=&\sum_{\alpha=1}^{\mathcal{L}} \sum_{\substack{i=1\\(i,\alpha)\in S}}^{N} \sum_{\substack{j=1\\(j,\alpha)\in S}}^{i-1} \sum_{\beta=1}^{\mathcal{L}} \sum_{\substack{k=1\\(k,\beta)\in S}}^{N} \sum_{\substack{r=1\\(r,\beta)\in S}}^{k-1} \left(\mathrm{E}[C^{\mathrm{con}}_{ij\alpha} C^{\mathrm{con}}_{kr\beta}] - C_{ij\alpha} C_{kr\beta}\right)\\
=&\sum_{\alpha=1}^{\mathcal{L}} \sum_{\substack{i=1\\(i,\alpha)\in S}}^{N} \sum_{\substack{j=1\\(j,\alpha)\in S}}^{i-1} \sum_{\beta=1}^{\mathcal{L}} \sum_{\substack{k=1\\(k,\beta)\in S}}^{N} \sum_{\substack{r=1\\(r,\beta)\in S}}^{k-1} \left\{\mathrm{E}\left[\left(\frac{1}{L}\sum_{l=1}^{L} x_{il\alpha} x_{jl\alpha}\right)\left(\frac{1}{L}\sum_{l=1}^{L} x_{kl\beta} x_{rl\beta}\right)\right] - C_{ij\alpha} C_{kr\beta}\right\}\\
=&\sum_{\alpha=1}^{\mathcal{L}} \sum_{\substack{i=1\\(i,\alpha)\in S}}^{N} \sum_{\substack{j=1\\(j,\alpha)\in S}}^{i-1} \sum_{\beta=1}^{\mathcal{L}} \sum_{\substack{k=1\\(k,\beta)\in S}}^{N} \sum_{\substack{r=1\\(r,\beta)\in S}}^{k-1} \left(\frac{1}{L^2}\mathrm{E}\left[\sum_{l=1}^{L} \sum_{l'=1}^{L} x_{il\alpha} x_{jl\alpha} x_{kl'\beta} x_{rl'\beta}\right] - C_{ij\alpha} C_{kr\beta}\right),
\end{aligned} \tag{S23}$$

where L is the number of samples we draw from the N-variate multivariate normal distribution. Now, using the fact that different samples are independent, we obtain

$$
\begin{aligned}
&\frac{1}{L^2}\mathrm{E}\left[\sum_{l=1}^{L}\sum_{l'=1}^{L} x_{il\alpha}x_{jl\alpha}x_{kl'\beta}x_{rl'\beta}\right] - C_{ij\alpha}C_{kr\beta} \\
=&\frac{1}{L^2}\sum_{l=1}^{L}\sum_{\substack{l'=1\\ l'\neq l}}^{L}\mathrm{E}[x_{il\alpha}x_{jl\alpha}x_{kl'\beta}x_{rl'\beta}] + \frac{1}{L^2}\sum_{l=1}^{L}\mathrm{E}[x_{il\alpha}x_{jl\alpha}x_{kl\beta}x_{rl\beta}] - C_{ij\alpha}C_{kr\beta} \\
=&\frac{1}{L^2}\sum_{l=1}^{L}\mathrm{E}[x_{il\alpha}x_{jl\alpha}]\cdot\sum_{\substack{l'=1\\ l'\neq l}}^{L}\mathrm{E}[x_{kl'\beta}x_{rl'\beta}] + \frac{1}{L^2}\sum_{l=1}^{L}\mathrm{E}[x_{il\alpha}x_{jl\alpha}x_{kl\beta}x_{rl\beta}] - C_{ij\alpha}C_{kr\beta} \\
=&\frac{1}{L^2}\cdot LC_{ij\alpha}\cdot(L-1)C_{kr\beta} + \frac{1}{L^2}\sum_{l=1}^{L}\mathrm{E}[x_{il\alpha}x_{jl\alpha}x_{kl\beta}x_{rl\beta}] - C_{ij\alpha}C_{kr\beta} \\
=&\frac{(L-1)}{L}C_{ij\alpha}C_{kr\beta} + \frac{1}{L^2}\sum_{l=1}^{L}\mathrm{E}[x_{il\alpha}x_{jl\alpha}x_{kl\beta}x_{rl\beta}] - C_{ij\alpha}C_{kr\beta} \\
=&-\frac{1}{L}C_{ij\alpha}C_{kr\beta} + \frac{1}{L^2}\sum_{l=1}^{L}\mathrm{E}[x_{il\alpha}x_{jl\alpha}x_{kl\beta}x_{rl\beta}]. \qquad \text{(S24)}
\end{aligned}
$$

By substituting Eq. (S24) into Eq. (S23), using the fact that the covariance matrices C_α and C_β are independent when $\beta \neq \alpha$, and using Isserlis' Theorem [12], we obtain

$$
\begin{aligned}
\mathrm{Var}\left[\sum_{\alpha=1}^{\mathcal{L}} \sum_{\substack{i=1\\(i,\alpha)\in S}}^{N} \sum_{\substack{j=1\\(j,\alpha)\in S}}^{i-1} C_{ij\alpha}^{\mathrm{con}}\right] =& \sum_{\alpha=1}^{\mathcal{L}} \sum_{\substack{i=1\\(i,\alpha)\in S}}^{N} \sum_{\substack{j=1\\(j,\alpha)\in S}}^{i-1} \sum_{\beta=1}^{\mathcal{L}} \sum_{\substack{k=1\\(k,\beta)\in S}}^{N} \sum_{\substack{r=1\\(r,\beta)\in S}}^{k-1} \left(-\frac{1}{L} C_{ij\alpha} C_{kr\beta} + \frac{1}{L^2} \sum_{l=1}^{L} \mathrm{E}[x_{il\alpha} x_{jl\alpha} x_{kl\beta} x_{rl\beta}]\right) \\
=& \sum_{\alpha=1}^{\mathcal{L}} \sum_{\substack{i=1\\(i,\alpha)\in S}}^{N} \sum_{\substack{j=1\\(j,\alpha)\in S}}^{i-1} \sum_{\substack{\beta=1\\\beta\neq\alpha}}^{\mathcal{L}} \sum_{\substack{k=1\\(k,\beta)\in S}}^{N} \sum_{\substack{r=1\\(r,\beta)\in S}}^{k-1} \left(-\frac{1}{L} C_{ij\alpha} C_{kr\beta} + \frac{1}{L^2} \sum_{l=1}^{L} \mathrm{E}[x_{il\alpha} x_{jl\alpha} x_{kl\beta} x_{rl\beta}]\right) \\
&+ \sum_{\alpha=1}^{\mathcal{L}} \sum_{\substack{i=1\\(i,\alpha)\in S}}^{N} \sum_{\substack{j=1\\(j,\alpha)\in S}}^{i-1} \sum_{\substack{k=1\\(k,\alpha)\in S}}^{N} \sum_{\substack{r=1\\(r,\alpha)\in S}}^{k-1} \left(-\frac{1}{L} C_{ij\alpha} C_{kr\alpha} + \frac{1}{L^2} \sum_{l=1}^{L} \mathrm{E}[x_{il\alpha} x_{jl\alpha} x_{kl\alpha} x_{rl\alpha}]\right) \\
=& -\frac{1}{L}\left(\sum_{\alpha=1}^{\mathcal{L}} \sum_{\substack{i=1\\(i,\alpha)\in S}}^{N} \sum_{\substack{j=1\\(j,\alpha)\in S}}^{i-1} C_{ij\alpha}\right)\left(\sum_{\substack{\beta=1\\\beta\neq\alpha}}^{\mathcal{L}} \sum_{\substack{k=1\\(k,\beta)\in S}}^{N} \sum_{\substack{r=1\\(r,\beta)\in S}}^{k-1} C_{kr\beta}\right) \\
&+ \frac{1}{L^2} \sum_{l=1}^{L}\left(\sum_{\alpha=1}^{\mathcal{L}} \sum_{\substack{i=1\\(i,\alpha)\in S}}^{N} \sum_{\substack{j=1\\(j,\alpha)\in S}}^{i-1} \mathrm{E}[x_{il\alpha} x_{jl\alpha}]\right)\left(\sum_{\substack{\beta=1\\\beta\neq\alpha}}^{\mathcal{L}} \sum_{\substack{k=1\\(k,\beta)\in S}}^{N} \sum_{\substack{r=1\\(r,\beta)\in S}}^{k-1} \mathrm{E}[x_{kl\beta} x_{rl\beta}]\right) \\
&- \frac{1}{L} \sum_{\alpha=1}^{\mathcal{L}}\left(\sum_{\substack{i=1\\(i,\alpha)\in S}}^{N} \sum_{\substack{j=1\\(j,\alpha)\in S}}^{i-1} C_{ij\alpha}\right)\left(\sum_{\substack{k=1\\(k,\alpha)\in S}}^{N} \sum_{\substack{r=1\\(r,\alpha)\in S}}^{k-1} C_{kr\alpha}\right) \\
&+ \frac{1}{L^2} \sum_{l=1}^{L}\left\{\sum_{\alpha=1}^{\mathcal{L}} \sum_{\substack{i=1\\(i,\alpha)\in S}}^{N} \sum_{\substack{j=1\\(j,\alpha)\in S}}^{i-1} \sum_{\substack{k=1\\(k,\alpha)\in S}}^{N} \sum_{\substack{r=1\\(r,\alpha)\in S}}^{k-1} (\mathrm{E}[x_{il\alpha} x_{jl\alpha}] \cdot \mathrm{E}[x_{kl\alpha} x_{rl\alpha}]\right. \\
&\left. + \mathrm{E}[x_{il\alpha} x_{kl\alpha}] \cdot \mathrm{E}[x_{jl\alpha} x_{rl\alpha}] + \mathrm{E}[x_{il\alpha} x_{rl\alpha}] \cdot \mathrm{E}[x_{jl\alpha} x_{kl\alpha}])\right\} \\
=& -\frac{1}{L}\left(\sum_{\alpha=1}^{\mathcal{L}} \sum_{\substack{i=1\\(i,\alpha)\in S}}^{N} \sum_{\substack{j=1\\(j,\alpha)\in S}}^{i-1} C_{ij\alpha}\right)\left(\sum_{\substack{\beta=1\\\beta\neq\alpha}}^{\mathcal{L}} \sum_{\substack{k=1\\(k,\beta)\in S}}^{N} \sum_{\substack{r=1\\(r,\beta)\in S}}^{k-1} C_{kr\beta}\right) \\
&+ \frac{1}{L^2} \cdot L \left(\sum_{\alpha=1}^{\mathcal{L}} \sum_{\substack{i=1\\(i,\alpha)\in S}}^{N} \sum_{\substack{j=1\\(j,\alpha)\in S}}^{i-1} C_{ij\alpha}\right)\left(\sum_{\substack{\beta=1\\\beta\neq\alpha}}^{\mathcal{L}} \sum_{\substack{k=1\\(k,\beta)\in S}}^{N} \sum_{\substack{r=1\\(r,\beta)\in S}}^{k-1} C_{kr\beta}\right) \\
&- \frac{1}{L} \sum_{\alpha=1}^{\mathcal{L}}\left(\sum_{\substack{i=1\\(i,\alpha)\in S}}^{N} \sum_{\substack{j=1\\(j,\alpha)\in S}}^{i-1} C_{ij\alpha}\right)\left(\sum_{\substack{k=1\\(k,\alpha)\in S}}^{N} \sum_{\substack{r=1\\(r,\alpha)\in S}}^{k-1} C_{kr\alpha}\right) \\
&+ \frac{1}{L^2} \cdot L \left[\sum_{\alpha=1}^{\mathcal{L}} \sum_{\substack{i=1\\(i,\alpha)\in S}}^{N} \sum_{\substack{j=1\\(j,\alpha)\in S}}^{i-1} \sum_{\substack{k=1\\(k,\alpha)\in S}}^{N} \sum_{\substack{r=1\\(r,\alpha)\in S}}^{k-1} (C_{ij\alpha} C_{kr\alpha} + C_{ik\alpha} C_{jr\alpha} + C_{ir\alpha} C_{jk\alpha})\right].
\end{aligned}
\tag{S25}
$$

By combining the first and third terms in Eq. (S25), we obtain

$$
\begin{aligned}
\mathrm{Var}\left[\sum_{\alpha=1}^{\mathcal{L}}\sum_{\substack{i=1\\(i,\alpha)\in S}}^{N}\sum_{\substack{j=1\\(j,\alpha)\in S}}^{i-1} C^{\mathrm{con}}_{ij\alpha}\right] =& -\frac{1}{L}\left(\sum_{\alpha=1}^{\mathcal{L}}\sum_{\substack{i=1\\(i,\alpha)\in S}}^{N}\sum_{\substack{j=1\\(j,\alpha)\in S}}^{i-1} C_{ij\alpha}\right)\left(\sum_{\beta=1}^{\mathcal{L}}\sum_{\substack{k=1\\(k,\beta)\in S}}^{N}\sum_{\substack{r=1\\(r,\beta)\in S}}^{k-1} C_{kr\beta}\right)\\
&+\frac{1}{L}\left(\sum_{\alpha=1}^{\mathcal{L}}\sum_{\substack{i=1\\(i,\alpha)\in S}}^{N}\sum_{\substack{j=1\\(j,\alpha)\in S}}^{i-1} C_{ij\alpha}\right)\left(\sum_{\substack{\beta=1\\\beta\neq\alpha}}^{\mathcal{L}}\sum_{\substack{k=1\\(k,\beta)\in S}}^{N}\sum_{\substack{r=1\\(r,\beta)\in S}}^{k-1} C_{kr\beta}\right)\\
&+\frac{1}{L}\left[\sum_{\alpha=1}^{\mathcal{L}}\sum_{\substack{i=1\\(i,\alpha)\in S}}^{N}\sum_{\substack{j=1\\(j,\alpha)\in S}}^{i-1}\sum_{\substack{k=1\\(k,\alpha)\in S}}^{N}\sum_{\substack{r=1\\(r,\alpha)\in S}}^{k-1}\left(C_{ij\alpha}C_{kr\alpha}+C_{ik\alpha}C_{jr\alpha}+C_{ir\alpha}C_{jk\alpha}\right)\right]\\
=& -\frac{1}{L}\left(\sum_{\alpha=1}^{\mathcal{L}}\sum_{\substack{i=1\\(i,\alpha)\in S}}^{N}\sum_{\substack{j=1\\(j,\alpha)\in S}}^{i-1} C_{ij\alpha}\right)^2\\
&+\frac{1}{L}\left(\sum_{\alpha=1}^{\mathcal{L}}\sum_{\substack{i=1\\(i,\alpha)\in S}}^{N}\sum_{\substack{j=1\\(j,\alpha)\in S}}^{i-1} C_{ij\alpha}\right)\left(\sum_{\substack{\beta=1\\\beta\neq\alpha}}^{\mathcal{L}}\sum_{\substack{k=1\\(k,\beta)\in S}}^{N}\sum_{\substack{r=1\\(r,\beta)\in S}}^{k-1} C_{kr\beta}\right)\\
&+\frac{1}{L}\left(\sum_{\alpha=1}^{\mathcal{L}}\sum_{\substack{i=1\\(i,\alpha)\in S}}^{N}\sum_{\substack{j=1\\(j,\alpha)\in S}}^{i-1}\sum_{\substack{k=1\\(k,\alpha)\in S}}^{N}\sum_{\substack{r=1\\(r,\alpha)\in S}}^{k-1} C_{ij\alpha}C_{kr\alpha}\right)\\
&+\frac{1}{L}\left[\sum_{\alpha=1}^{\mathcal{L}}\sum_{\substack{i=1\\(i,\alpha)\in S}}^{N}\sum_{\substack{j=1\\(j,\alpha)\in S}}^{i-1}\sum_{\substack{k=1\\(k,\alpha)\in S}}^{N}\sum_{\substack{r=1\\(r,\alpha)\in S}}^{k-1}\left(C_{ik\alpha}C_{jr\alpha}+C_{ir\alpha}C_{jk\alpha}\right)\right]\\
=& -\frac{1}{L}\left(\sum_{\alpha=1}^{\mathcal{L}}\sum_{\substack{i=1\\(i,\alpha)\in S}}^{N}\sum_{\substack{j=1\\(j,\alpha)\in S}}^{i-1} C_{ij\alpha}\right)^2+\frac{1}{L}\left(\sum_{\alpha=1}^{\mathcal{L}}\sum_{\substack{i=1\\(i,\alpha)\in S}}^{N}\sum_{\substack{j=1\\(j,\alpha)\in S}}^{i-1} C_{ij\alpha}\right)^2\\
&+\frac{1}{L}\left[\sum_{\alpha=1}^{\mathcal{L}}\sum_{\substack{i=1\\(i,\alpha)\in S}}^{N}\sum_{\substack{j=1\\(j,\alpha)\in S}}^{i-1}\sum_{\substack{k=1\\(k,\alpha)\in S}}^{N}\sum_{\substack{r=1\\(r,\alpha)\in S}}^{k-1}\left(C_{ik\alpha}C_{jr\alpha}+C_{ir\alpha}C_{jk\alpha}\right)\right]\\
=&\frac{1}{L}\left[\sum_{\alpha=1}^{\mathcal{L}}\sum_{\substack{i=1\\(i,\alpha)\in S}}^{N}\sum_{\substack{j=1\\(j,\alpha)\in S}}^{i-1}\sum_{\substack{k=1\\(k,\alpha)\in S}}^{N}\sum_{\substack{r=1\\(r,\alpha)\in S}}^{k-1}\left(C_{ik\alpha}C_{jr\alpha}+C_{ir\alpha}C_{jk\alpha}\right)\right]. \qquad \text{(S26)}
\end{aligned}
$$

Text D. Graphical lasso

For multivariate Gaussian distributions, a zero in the precision matrix (i.e., inverse covariance matrix) is equivalent to conditional independence of two variables, which one can relate to the absence of an edge in the network. Therefore, it is natural to use the non-zero entries of the estimated precision matrix to determine the edges [13]. However, when the number of variables is larger than the number of samples, the empirical covariance matrix is not full rank. In this case, the empirical covariance matrix is singular, meaning that its condition number is infinite, so estimating the precision matrix becomes difficult [14]. The graphical

lasso addresses this problem by regularizing the maximum likelihood estimator with a lasso penalty enforcing sparsity [14–16].

Let $\vec{y}$ be a p-variate Gaussian random column vector, with distribution $\mathcal{N}(\mu, C)$, where μ is the p-dimensional mean vector and C is the $p \times p$ covariance matrix. Given n independently drawn samples $\{\vec{y_1}, \ldots, \vec{y_n}\}$ of this random vector, the sample covariance matrix can be written as

$$\hat{C} = \frac{1}{n-1} \sum_{k=1}^{n} (\vec{y_k} - \hat{\mu})(\vec{y_k} - \hat{\mu})^\top, \tag{S27}$$

where $\hat{\mu} = \frac{1}{n} \sum_{k=1}^{n} \vec{y_k}$, and $^\top$ represents the transposition. Let the inverse covariance matrix be denoted as $C^{-1} = \Theta$. We consider a generalized ℓ_1 regularization given by $\lambda \sum_{i=1}^{p} \sum_{j=1}^{i-1} |\Theta_{ij}|$, where λ is the penalizing parameter. Then, the problem is to maximize the lasso regularized log-likelihood to obtain the graphical lasso estimator, i.e.,

$$\Theta^* = \arg\min_{\Theta \succ 0} \left\{ -\log\det\Theta + \mathrm{tr}(\hat{C}\Theta) + \lambda \sum_{i=1}^{p} \sum_{j=1}^{i-1} |\Theta_{ij}| \right\}, \tag{S28}$$

where $\Theta \succ 0$ signifies that Θ is a positive definite matrix [14].

Using the TPM data for each of the four tissues separately, we first calculated the 203×203 empirical covariance matrix. Then, we applied the GraphicalLassoCV function from the Python package scikit-learn version 1.0.2 [17, 18] to estimate a precision matrix, which is a sparsified co-expression network, for each tissue. This graphical lasso algorithm incorporates a cross-validated choice of the ℓ_1 penalty. To simplify analysis, we regard the generated networks as unsigned and unweighted network. The results for the unsigned weighted networks are similar to those for the unsigned unweighted networks, as we will show.

Text E. Z scores for the average distance between pairs of genes on each chromosome separately in each community

We show in Table B the Z scores for the average distance between pairs of genes on each chromosome and each community with $\gamma = 1$. We show the corresponding results with $\gamma = 3$ in Table C. We only calculated the Z scores for the chromosome-community pairs with at least 3 genes. In these tables, N/A implies that either there are less than 3 genes, or the standard deviation of the average distance is equal to 0 because every random selection of genes is the same gene set. The latter event occurs when all the genes on that chromosome are associated with the same community.

Table B. Z scores for the average distance between pairs of genes on each chromosome and each significant community detected with $\gamma = 1$. Comm. denotes community, and Chr denotes chromosome.

$\gamma = 1$					
	Comm.				
Chr	1	2	3	4	5
1	−2.093	−1.526	−0.122	−1.466	1.272
2	N/A	−0.767	0.061	2.129	−0.725
3	N/A	N/A	0.113	1.101	N/A
4	N/A	N/A	N/A	−0.110	−2.573
5	N/A	N/A	N/A	N/A	N/A
6	0.606	N/A	N/A	0.564	−0.065
7	N/A	−1.938	−1.842	0.968	−0.302
8	N/A	N/A	N/A	N/A	N/A
9	N/A	N/A	N/A	−0.722	N/A
10	N/A	−2.336	0.413	N/A	N/A
11	N/A	N/A	−2.156	0.077	0.454
12	N/A	N/A	−1.376	−0.905	−0.260
13	N/A	N/A	N/A	N/A	N/A
14	N/A	N/A	0.438	N/A	0.180
15	N/A	N/A	N/A	N/A	N/A
16	−0.091	0.737	0.100	N/A	N/A
17	1.535	N/A	−2.160	−0.579	−0.787
18	N/A	N/A	N/A	N/A	N/A
19	0.235	N/A	−0.521	1.592	1.894
20	N/A	N/A	N/A	N/A	0.791
21	N/A	N/A	N/A	N/A	N/A
22	N/A	N/A	N/A	N/A	−1.780
X	N/A	N/A	N/A	N/A	N/A
Y	N/A	N/A	N/A	N/A	N/A
M	N/A	N/A	N/A	N/A	−2.061

Text F. Results of the gene set enrichment analysis

We show the two most significant GO:BP and HP results from g:Profiler for the top 50 highly expressed genes out of the 203 genes in the network in each tissue in Table D. See [19] for the entire output from g:Profiler, i.e., the list of all significant GO:BP and HP results, for the top 50 genes in pancreas; see [20] for the top 50 genes in salivary gland; see [21] for the top 50 genes in mammary gland; and see [22] for the top 50 genes in skin.

We show the two most significant GO:BP and HP results from g:Profiler for each community in the partition of the multilayer correlation matrix with $\gamma = 3$ in Table E. See [23] for the entire output from g:Profiler, i.e., the list of all significant GO:BP and HP results, for the genes in community 1; see [24] for community 2; see [25] for community 3; see [26] for community 4; see [27] for community 5; see [28] for community 6; and see [29] for community 7.

We show the two most significant GO:BP and HP results from g:Profiler for the top 50 highly connected genes (i.e., top 50 hub genes) out of the 203 genes in the network in each tissue in Table F. See [30] for the entire output from g:Profiler, i.e., the list of all significant GO:BP and HP results, for the top 50 genes in pancreas; see [31] for the top 50 genes in salivary gland; see [32] for the top 50 genes in mammary gland; and see [33] for the top 50 genes in skin.

Table C. Z scores for the average distance between pairs of genes on each chromosome and each significant community detected with $\gamma = 3$. Comm. denotes community, and Chr denotes chromosome.

$\gamma = 3$							
	Comm.						
Chr	1	2	3	4	5	6	7
1	−2.808	0.116	−0.447	N/A	−0.482	N/A	N/A
2	−0.671	N/A	N/A	N/A	2.094	−2.220	N/A
3	N/A	N/A	N/A	N/A	N/A	N/A	N/A
4	N/A	N/A	N/A	N/A	−1.993	N/A	N/A
5	N/A	N/A	N/A	N/A	N/A	N/A	N/A
6	N/A	0.573	N/A	N/A	N/A	N/A	N/A
7	−1.643	N/A	N/A	N/A	−2.616	N/A	N/A
8	N/A	N/A	N/A	N/A	N/A	N/A	N/A
9	N/A	N/A	N/A	N/A	N/A	N/A	N/A
10	N/A	N/A	N/A	N/A	0.293	N/A	N/A
11	N/A	N/A	N/A	N/A	−2.902	N/A	N/A
12	N/A	1.729	N/A	N/A	−0.577	N/A	N/A
13	N/A	N/A	N/A	N/A	N/A	N/A	N/A
14	N/A	N/A	N/A	N/A	N/A	−1.756	N/A
15	N/A	N/A	N/A	N/A	N/A	N/A	N/A
16	−0.416	0.920	N/A	N/A	1.016	N/A	N/A
17	N/A	1.639	N/A	N/A	−2.175	N/A	N/A
18	N/A	N/A	N/A	N/A	N/A	N/A	N/A
19	N/A	−1.142	N/A	N/A	−0.179	N/A	N/A
20	N/A	N/A	N/A	N/A	N/A	N/A	N/A
21	N/A	N/A	N/A	N/A	N/A	N/A	N/A
22	N/A	N/A	N/A	N/A	N/A	N/A	N/A
X	N/A	N/A	N/A	N/A	N/A	N/A	N/A
Y	N/A	N/A	N/A	N/A	N/A	N/A	N/A
M	N/A	N/A	N/A	N/A	N/A	N/A	N/A

Text G. Tissue-specific hub genes versus gene communities

To compare the overlap of the top 50 highly connected genes in each tissue and the gene communities identified by our algorithm with $\gamma = 3$, we calculate the Jaccard index for each pair of the set of the 50 most connected genes in one of the four tissues and one of the seven gene communities identified by our algorithm. The Jaccard index is equal to 1 if the two sets perfectly overlap and 0 if the two sets are disjoint. We show the $4 \times 7 = 28$ Jaccard index values in Fig B. The largest Jaccard index is 0.324, revealing the lack of notable similarity between all of the 28 pairs of gene sets. Therefore, we conclude that our multilayer community detection method uncovers sets of genes that are different from top hub genes in each layer.

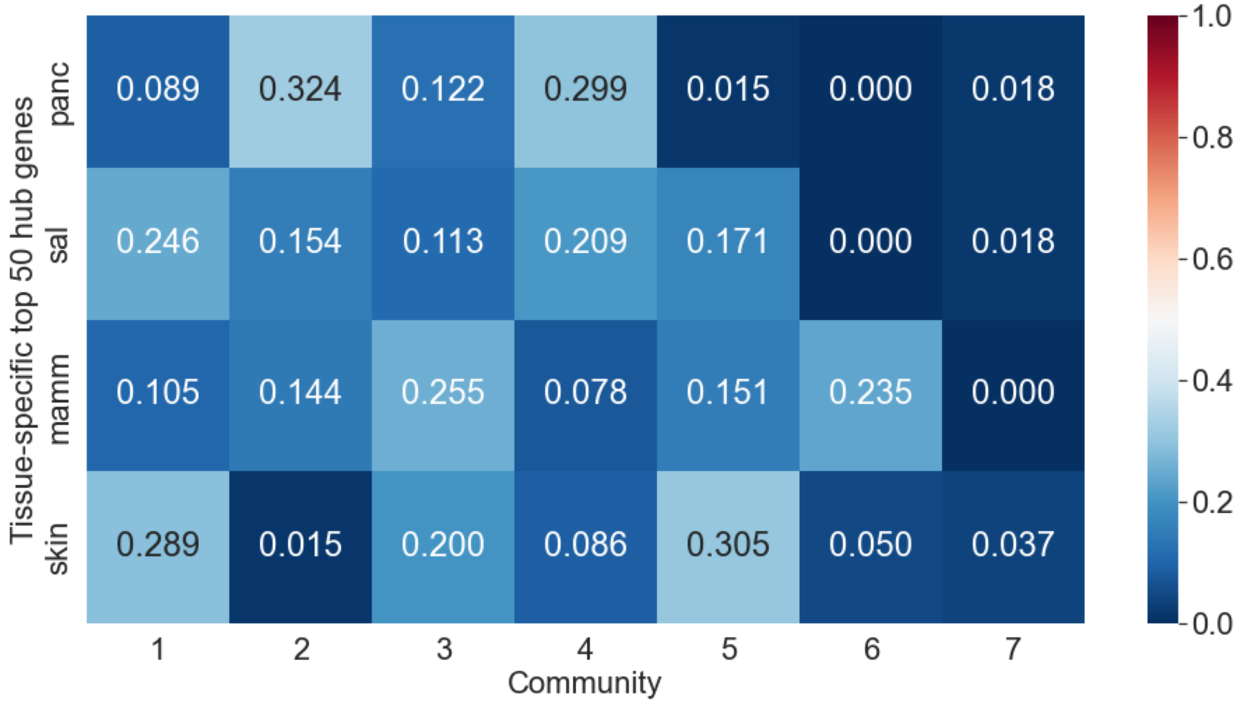

Fig B. Jaccard index between the set of tissue-specific hub genes and the set of genes in a community. Each row corresponds to the top 50 hub genes in each layer (i.e., tissue), where "panc" denotes pancreas, "sal" denotes salivary gland, "mamm" denotes mammary gland, and "skin" denotes skin (not sun exposed). Each column corresponds to a community identified with $\gamma = 3$.

Table D. Results of the gene set enrichment analysis for the top 50 highly expressed genes out of the 203 genes in the network in each tissue.

Tissue	Top significant terms from GO:BP	p value	Top significant terms from HP	p value
pancreas	oxidative phosphorylation	$6.39 \cdot 10^{-15}$	recurrent pancreatitis	$1.44 \cdot 10^{-22}$
	aerobic electron transport chain	$4.69 \cdot 10^{-14}$	mitochondrial inheritance	$1.79 \cdot 10^{-20}$
salivary gland	oxidative phosphorylation	$7.02 \cdot 10^{-16}$	mitochondrial inheritance	$6.33 \cdot 10^{-21}$
	aerobic electron transport chain	$7.37 \cdot 10^{-15}$	centrocecal scotoma	$3.23 \cdot 10^{-20}$
mammary gland	oxidative phosphorylation	$6.35 \cdot 10^{-15}$	mitochondrial inheritance	$9.67 \cdot 10^{-20}$
	mitochondrial ATP synthesis coupled electron transport	$4.77 \cdot 10^{-14}$	centrocecal scotoma	$3.63 \cdot 10^{-19}$
skin	oxidative phosphorylation	$7.28 \cdot 10^{-15}$	mitochondrial inheritance	$5.06 \cdot 10^{-19}$
	aerobic electron transport chain	$5.35 \cdot 10^{-14}$	centrocecal scotoma	$1.59 \cdot 10^{-18}$

Table E. Results of the gene set enrichment analysis for the communities of the multilayer correlation matrix with $\gamma = 3$. Comm. denotes community.

Comm.	Top significant terms from GO:BP	p value	Top significant terms from HP	p value
1	oxidative phosphorylation	$5.90 \cdot 10^{-11}$	recurrent pancreatitis	$1.78 \cdot 10^{-19}$
	aerobic electron transport chain	$1.05 \cdot 10^{-10}$	mitochondrial inheritance	$1.06 \cdot 10^{-17}$
2	keratinocyte differentiation	$8.26 \cdot 10^{-25}$	palmoplantar keratoderma	$3.05 \cdot 10^{-7}$
	epidermis development	$1.90 \cdot 10^{-24}$	hyperkeratosis	$3.05 \cdot 10^{-7}$
3	retina homeostasis	$2.52 \cdot 10^{-10}$	leber optic atophy	$6.65 \cdot 10^{-15}$
	oxidative phosphorylation	$2.23 \cdot 10^{-9}$	mitochondrial inheritance	$6.65 \cdot 10^{-15}$
4	skin development	$3.23 \cdot 10^{-13}$	alopecia	$8.30 \cdot 10^{-5}$
	intermediate filament organization	$2.64 \cdot 10^{-12}$	nail dystrophy	$8.30 \cdot 10^{-5}$
5	keratinization	$1.95 \cdot 10^{-19}$	palmoplantar blistering	$1.71 \cdot 10^{-6}$
	epidermis development	$1.95 \cdot 10^{-19}$	palmoplantar keratoderma	$2.02 \cdot 10^{-5}$
6	positive regulation of respiratory burst	$5.36 \cdot 10^{-8}$	N/A	N/A
	regulation of respiratory burst	$2.60 \cdot 10^{-7}$	N/A	N/A
7	adaptive thermogenesis	$1.73 \cdot 10^{-2}$	N/A	N/A
	fatty acid biosynthesis process	$1.73 \cdot 10^{-2}$	N/A	N/A

Table F. Results of the gene set enrichment analysis for the top 50 highly connected genes out of the 203 genes in the single-layer network of each tissue.

Tissue	Top significant terms from GO:BP	p value	Top significant terms from HP	p value
pancreas	cytoplasmic translation	$1.68 \cdot 10^{-10}$	mutism	$1.77 \cdot 10^{-3}$
	sequestering of metal ion	$2.49 \cdot 10^{-8}$	arterial rupture	$1.93 \cdot 10^{-3}$
salivary gland	digestion	$3.05 \cdot 10^{-8}$	pancreatic calcification	$5.28 \cdot 10^{-9}$
	cytoplasmic translation	$4.52 \cdot 10^{-5}$	pancreatic pseudocyst	$5.66 \cdot 10^{-8}$
mammary gland	antibacterial humoral response	$3.23 \cdot 10^{-10}$	nail dystrophy	$9.67 \cdot 10^{-5}$
	defense response to bacterium	$3.36 \cdot 10^{-8}$	palmoplantar blistering	$4.37 \cdot 10^{-4}$
skin	digestion	$1.59 \cdot 10^{-8}$	pancreatic calcification	$4.68 \cdot 10^{-6}$
	proteolysis	$4.89 \cdot 10^{-6}$	recurrent pancreatitis	$4.82 \cdot 10^{-5}$

Supplementary References

1. Park J, Newman MEJ. Origin of degree correlations in the Internet and other networks. Physical Review E. 2003;68(2):026112.

2. Catanzaro M, Boguñá M, Pastor-Satorras R. Generation of uncorrelated random scale-free networks. Physical Review E. 2005;71(2):027103.

3. Maslov S, Sneppen K, Zaliznyak A. Detection of topological patterns in complex networks: correlation profile of the internet. Physica A. 2004;333:529–540.

4. Garlaschelli D, Loffredo MI. Generalized Bose-Fermi statistics and structural correlations in weighted networks. Physical Review Letters. 2009;102(3):038701.

5. Park J, Newman MEJ. Statistical mechanics of networks. Physical Review E. 2004;70(6):066117.

6. Cimini G, Squartini T, Saracco F, Garlaschelli D, Gabrielli A, Caldarelli G. The statistical physics of real-world networks. Nature Reviews Physics. 2019;1:58–71.

7. Vallarano N, Bruno M, Marchese E, Trapani G, Saracco F, Cimini G, et al. Fast and scalable likelihood maximization for Exponential Random Graph Models with local constraints. Scientific Reports. 2021;11:15227.

8. Radicchi F, Castellano C, Cecconi F, Loreto V, Parisi D. Defining and identifying communities in networks. Proceedings of the National Academy of Sciences of the United States of America. 2004;101(9):2658–2663.

9. Yang J, Leskovec J. Defining and evaluating network communities based on ground-truth. Knowledge and Information Systems. 2015;42(1):181–213.

10. Wang YH. On the number of successes in independent trials. Statistica Sinica. 1993;3(2):295–312.

11. Shi J, Malik J. Normalized cuts and image segmentation. IEEE Transactions on Pattern Analysis and Machine Intelligence. 2000;22(8):888–905.

12. Isserlis L. On a formula for the product-moment coefficient of any order of a normal frequency distribution in any number of variables. Biometrika. 1918;12(1/2):134–139.

13. Hsieh CJ, Sustik MA, Dhillon IS, Ravikumar P. QUIC: Quadratic Approximation for Sparse Inverse Covariance Estimation. Journal of Machine Learning Research. 2014;15:2911–2947.

14. Friedman J, Hastie T, Tibshirani R. Sparse inverse covariance estimation with the graphical lasso. Biostatistics. 2008;9(3):432–441.

15. Meinshausen N, Bühlmann P. High-dimensional graphs and variable selection with the Lasso. Annals of Statistics. 2006;34(3):1436–1462.

16. Yuan M, Lin Y. Model selection and estimation in the Gaussian graphical model. Biometrika. 2007;94(1):19–35.

17. Pedregosa F, Varoquaux G, Gramfort A, Michel V, Thirion B, Grisel O, et al. Scikit-learn: machine learning in Python. Journal of Machine Learning Research. 2011;12:2825–2830.

18. scikit-learn package. Version 1.0.2 [software]. 2021 Jan [downloaded 2022 May 18]. Available from: `https://scikit-learn.org/stable/install.html`.

19. g:Profiler g:GOSt (pancreas, highly expressed). 2023 Apr 27. Available from: `https://biit.cs.ut.ee/gplink/l/QbFURpSESM`.

20. g:Profiler g:GOSt (salivary gland, highly expressed). 2023 Apr 27. Available from: `https://biit.cs.ut.ee/gplink/l/ajM--6FRTY`.

21. g:Profiler g:GOSt (mammary gland, highly expressed). 2023 Apr 27. Available from: https://biit.cs.ut.ee/gplink/l/aDH27OkWTD.

22. g:Profiler g:GOSt (skin, highly expressed). 2023 Apr 27. Available from: https://biit.cs.ut.ee/gplink/l/F2kZch7qRG.

23. g:Profiler g:GOSt (community 1). 2023 Apr 27. Available from: https://biit.cs.ut.ee/gplink/l/TLbKa39zRU.

24. g:Profiler g:GOSt (community 2). 2023 Apr 27. Available from: https://biit.cs.ut.ee/gplink/l/OueSgf4HRJ.

25. g:Profiler g:GOSt (community 3). 2023 Apr 27. Available from: https://biit.cs.ut.ee/gplink/l/T21OqL34SQ.

26. g:Profiler g:GOSt (community 4). 2023 Apr 27. Available from: https://biit.cs.ut.ee/gplink/l/6p4rYKFtQE.

27. g:Profiler g:GOSt (community 5). 2023 Apr 27. Available from: https://biit.cs.ut.ee/gplink/l/JGyRZKeZQf.

28. g:Profiler g:GOSt (community 6). 2023 Apr 27. Available from: https://biit.cs.ut.ee/gplink/l/-xMfgdJbSo.

29. g:Profiler g:GOSt (community 7). 2023 Apr 27. Available from: https://biit.cs.ut.ee/gplink/l/eCuRxFu4QT.

30. g:Profiler g:GOSt (pancreas, highly connected). 2023 Jul 23. Available from: https://biit.cs.ut.ee/gplink/l/6sZ_-mTgSo.

31. g:Profiler g:GOSt (salivary gland, highly connected). 2023 Jul 23. Available from: https://biit.cs.ut.ee/gplink/l/u-KDytxVTJ.

32. g:Profiler g:GOSt (mammary gland, highly connected). 2023 Jul 23. Available from: https://biit.cs.ut.ee/gplink/l/gnrpFfIYT9.

33. g:Profiler g:GOSt (skin, highly connected). 2023 Jul 23. Available from: https://biit.cs.ut.ee/gplink/l/KGCZRB-mTk.
