# Supplementary material for: Gene communities in co-expression networks across different tissues
Source: ArXiv. 2023 May 22:arXiv:2305.12963v1. Preprint. [Version 1] (PMC10246089)
Supplement: Supplementary file 1 [file NIHPP2305.12963v1-supplement-1.pdf]

Supplementary Materials for:

Gene communities in co-expression networks across different tissues

Madison Russell, Marie Saitou, Omer Gokcumen, Naoki Masuda

S1 Significance of communities detected in general multilayer networks

S1.1 Null model

We assume undirected and unweighted networks. A common choice for a null model is the configuration model, in which the degree sequence $\{k_i\}_{i=1}^N$ is specified, and an edge is laid between nodes i and j with probability

$$p_{ij} = \frac{k_i k_j}{2M}, \tag{S1}$$

where k_i is the degree of the ith node in the original network. However, we avoid the configuration model for two reasons. First, by imposing the value of $k_1, \ldots, k_N$, the stochastic generation of edges sharing a node is not independent of each other. For example, if k_1 is small and we have generated edge $(1, 2)$ with probability p_{12}, then edge $(1, 3)$ is generated with a probability smaller than p_{13}. This type of correlation makes it difficult to analytically derive the quality measure of individual communities that requires the count the edges sharing a node. Second, large $k_i k_j$ values can yield $p_{ij} > 1$ [1]. One could set a structural cutoff degree $k_{\max}$ to be of the order of $\sqrt{N}$ to enforce the constraint $p_{ij} < 1$ [2]. However, it is often the case that the largest degree in an empirical network far exceeds this structural cutoff value [3, 4].

Instead of the configuration model, we use an exponential random graph model (ERGM) as the null model. Instead of the exact degree, we fix the expected degree of each ith node to k_i^*, the degree of the same node in the original network. Let Ω be an ensemble of networks with N nodes. Let $\vec{\theta} \equiv (\theta_1, \ldots, \theta_N)$ be the model parameters. The probability distribution of the adjacency matrix, $A = (A_{ij})$, that maximizes the Shannon entropy subject to the constraints

$$\sum_{A \in \Omega} P(A) k_i(A) = k_i^*, \tag{S2}$$

where $k_i(A)$ is the degree of the ith node in network A, and the normalization condition

$$\sum_{A \in \Omega} P(A) = 1 \tag{S3}$$

is

$$P(A|\vec{\theta}) = \prod_{i=1}^{N} \prod_{j=1}^{i-1} p_{ij}^{A_{ij}} (1 - p_{ij})^{(1 - A_{ij})}, \tag{S4}$$

where

$$p_{ij} = \frac{e^{-\theta_i - \theta_j}}{1 + e^{-\theta_i - \theta_j}} \tag{S5}$$

is the probability that nodes i and j are adjacent [5–7].

We infer the model parameters $\vec{\theta}$ by maximizing the associated log-likelihood function, i.e.,

$$\mathscr{L}(\vec{\theta}) \equiv \ln P(A^*|\vec{\theta}) = -\sum_{i=1}^{N} \theta_i k_i^* - \sum_{i=1}^{N} \sum_{j=1}^{i-1} \ln(1 + e^{-\theta_i - \theta_j}), \tag{S6}$$

where A^* is the adjacency matrix of the original network. One can derive Eq. (S6) from Eq. (S4).

We numerically determine θ_i's using a fixed point method implemented in the Python package NEMtropy [7]. We use NEMtropy to generate this so-called undirected binary configuration model (UBCM) [7]. We estimate the UBCM for each layer independently. Using the obtained multilayer UBCM as null model, we calculate the statistical significance of individual communities detected in the original multilayer network.

S1.2 Number of intralayer edges within each community

We use two measures to assess the quality of individual communities. The first measure is the number of intralayer edges within each community, which is essentially the same as X used in the main text for correlation matrices. Previous studies used the number of edges within a community in single layer networks [8,9], and we extend this quality measure to multilayer networks.

Let S be a set of nodes in a multilayer network. Let X_α be the number of edges within S in layer α and let X be the total number of intralayer edges within S, i.e.,

$$X = \sum_{\alpha=1}^{\mathcal{L}} X_\alpha. \tag{S7}$$

In the UBCM, the edges are independently laid. Therefore, X obeys the Poisson binomial distribution, which is the discrete probability distribution of a sum of independent Bernoulli trials that are not necessarily identically distributed [10].

Let $\theta_{i\alpha}$ be the UBCM parameter for node i in layer α. Using Eq. (S5) for each layer, we obtain the expectation of X as follows:

$$\mathrm{E}[X] = \sum_{\alpha=1}^{\mathcal{L}} \sum_{\substack{i=1\\(i,\alpha)\in S}}^{N} \sum_{\substack{j=1\\(j,\alpha)\in S}}^{i-1} \frac{e^{-\theta_{i\alpha}-\theta_{j\alpha}}}{1+e^{-\theta_{i\alpha}-\theta_{j\alpha}}}, \tag{S8}$$

where the summation is over all node pairs $(i,\alpha),(j,\alpha)$ in S. Note that we have excluded self-loops. The variance of X is equal to [10]

$$\mathrm{Var}[X] = \sum_{\alpha=1}^{\mathcal{L}} \sum_{\substack{i=1\\(i,\alpha)\in S}}^{N} \sum_{\substack{j=1\\(j,\alpha)\in S}}^{i-1} \left(1 - \frac{e^{-\theta_{i\alpha}-\theta_{j\alpha}}}{1+e^{-\theta_{i\alpha}-\theta_{j\alpha}}}\right) \frac{e^{-\theta_{i\alpha}-\theta_{j\alpha}}}{1+e^{-\theta_{i\alpha}-\theta_{j\alpha}}}. \tag{S9}$$

S1.3 Conductance of each community

The second quality measure is the conductance of each community. Let $G(V,E)$ be an undirected single-layer network, and consider a set of nodes $S \subseteq V$. Let

$$c_s = |\{(u,v) \in E : u \in S, v \notin S\}| \tag{S10}$$

be the number of edges on the boundary of S and

$$m_s = |\{(u,v) \in E : u \in S, v \in S\}| \tag{S11}$$

be the number of edges within S. Then, the conductance of S is given by [9]

$$\varphi(S) = \frac{c_s}{2m_s + c_s}. \tag{S12}$$

The conductance measures the fraction of the number of half-edges emanating from nodes in S that are connected to a half-edge emanating from a node outside S. Therefore, the conductance is small for a good community [11].

To define the conductance of a set of nodes S in a multilayer network with $\mathcal{L}$ layers, let Y_α be the number of edges on the boundary of S in layer α. We define the conductance of S by

$$\varphi(S) = \frac{\sum_{\alpha=1}^{\mathcal{L}} Y_\alpha}{\sum_{\alpha=1}^{\mathcal{L}}(2X_\alpha + Y_\alpha)} = \frac{Y}{2X+Y}, \tag{S13}$$

where Y is the number of intralayer edges on the boundary of S. Because the UBCM independently lays edges for different node pairs, Y as well as X obeys a Poisson binomial distribution. It should also be noted that X and Y are independent because they are calculated based on disjoint sets of node pairs.

We denote the set of intralayer node pairs within community S by

$$E_{\max}^{\text{within}} = \{(i, j, \alpha) : (i, \alpha) \in S, (j, \alpha) \in S, i < j\}. \tag{S14}$$

We note that the cardinality (i.e., number of elements) of $E_{\max}^{\text{within}}$ is equal to

$$x_{\max} \equiv \sum_{\alpha=1}^{L} \frac{N'_{S\alpha}(N'_{S\alpha} - 1)}{2}, \tag{S15}$$

where $N'_{S\alpha}$ is the number of nodes in S in layer α. Because X obeys the Poisson binomial distribution, the probability for X is

$$P_X(X = x) = \sum_{\overline{E} \subset E_{\max}^{\text{within}} \text{ s.t. } |\overline{E}| = x} \prod_{(i,j,\alpha) \in \overline{E}} p_{ij\alpha} \prod_{(i,j,\alpha) \in E_{\max}^{\text{within}} \setminus \overline{E}} (1 - p_{ij\alpha}), \tag{S16}$$

where

$$p_{ij\alpha} = \frac{e^{-\theta_{i\alpha} - \theta_{j\alpha}}}{1 + e^{-\theta_{i\alpha} - \theta_{j\alpha}}}, \tag{S17}$$

and x is the number of edges in S. Similarly, the probability for Y is

$$P_Y(Y = y) = \sum_{\overline{E} \subset E_{\max}^{\text{boundary}} \text{ s.t. } |\overline{E}| = y} \prod_{(i,j,\alpha) \in \overline{E}} p_{ij\alpha} \prod_{(i,j,\alpha) \in E_{\max}^{\text{boundary}} \setminus \overline{E}} (1 - p_{ij\alpha}), \tag{S18}$$

where

$$E_{\max}^{\text{boundary}} = \{(i, j, \alpha) : (i, \alpha) \in S, (j, \alpha) \notin S\}. \tag{S19}$$

Note that the cardinality of $E_{\max}^{\text{boundary}}$ is

$$y_{\max} = \sum_{\alpha=1}^{\mathcal{L}} N'_{S\alpha}(N - N'_{S\alpha}). \tag{S20}$$

Because X and Y are mutually independent, the expected value of the conductance of a set of nodes S in the multilayer network is given by

$$\mathrm{E}\left[\frac{Y}{2X + Y}\right] = \sum_{x=0}^{x_{\max}} \sum_{y=0}^{y_{\max}} \frac{y}{2x + y} P_X(X = x) P_Y(Y = y). \tag{S21}$$

The variance of the conductance of S is given by

$$\begin{aligned} \mathrm{Var}\left[\frac{Y}{2X + Y}\right] &= \sum_{x=0}^{x_{\max}} \sum_{y=0}^{y_{\max}} \left(\frac{y}{2x + y}\right)^2 P_X(X = x) P_Y(Y = y) \\ &\quad - \left[\sum_{x=0}^{x_{\max}} \sum_{y=0}^{y_{\max}} \frac{y}{2x + y} P_X(X = x) P_Y(Y = y)\right]^2. \end{aligned} \tag{S22}$$

In Eqs. (S21) and (S22), we set $\frac{y}{2x+y} = 1$ for $(x, y) = (0, 0)$.

S1.4 Results

We show in Table S1 the Z scores for the number of intralayer edges within each community and for the conductance of each community detected in the unweighted multilayer network obtained by graphical lasso with $\gamma = 1$ and $\gamma = 3$. For both partitions, all the communities with a Z score of N/A are composed of a single gene. Except these single-gene communities, for both $\gamma = 1$ and $\gamma = 3$, all other communities are statistically significant with a large positive Z score for the number of intralayer edges and a large negative Z score for the conductance.

Table S1: Z scores for the number of intralayer edges within each community and for the conductance of each community detected in the unweighted multilayer network obtained by graphical lasso with $\gamma = 1$ and $\gamma = 3$. Comm. denotes community and no. denotes "number of".

$\gamma = 1$			$\gamma = 3$		
Comm.	Z score for no. intralayer edges	Z score for the conductance	Comm.	Z score for no. intralayer edges	Z score for the conductance
1	26.787	−35.697	1	25.702	−31.396
2	43.706	−49.785	2	50.807	−60.543
3	41.245	−49.208	3	39.556	−42.975
4	49.956	−60.620	4	32.401	−34.112
5	42.446	−45.851	5	49.755	−52.415
6	43.054	−49.280	6	48.585	−52.584
7	34.795	−37.814	7	43.897	−46.904
8	29.940	−31.740	8	37.580	−40.672
9	57.664	−62.551	9	36.172	−37.726
10	N/A	N/A	10	30.597	−32.125
11	N/A	N/A	11	N/A	N/A
			12	N/A	N/A
			13	N/A	N/A
			14	N/A	N/A

S2 Derivation of the variance of the total intralayer weight for a community in a multilayer correlation matrix

To derive Eq. (13) in section 2.5, we start with

$$
\begin{aligned}
&\mathrm{Var}\left[\sum_{\alpha=1}^{\mathcal{L}} \sum_{\substack{i=1\\(i,\alpha)\in S}}^{N} \sum_{\substack{j=1\\(j,\alpha)\in S}}^{i-1} C_{ij\alpha}^{\mathrm{con}}\right] \\
=&\mathrm{E}\left[\left(\sum_{\alpha=1}^{\mathcal{L}} \sum_{\substack{i=1\\(i,\alpha)\in S}}^{N} \sum_{\substack{j=1\\(j,\alpha)\in S}}^{i-1} C_{ij\alpha}^{\mathrm{con}}\right)^2\right] - \left(\mathrm{E}\left[\sum_{\alpha=1}^{\mathcal{L}} \sum_{\substack{i=1\\(i,\alpha)\in S}}^{N} \sum_{\substack{j=1\\(j,\alpha)\in S}}^{i-1} C_{ij\alpha}^{\mathrm{con}}\right]\right)^2 \\
=&\mathrm{E}\left[\sum_{\alpha=1}^{\mathcal{L}} \sum_{\substack{i=1\\(i,\alpha)\in S}}^{N} \sum_{\substack{j=1\\(j,\alpha)\in S}}^{i-1} \sum_{\beta=1}^{\mathcal{L}} \sum_{\substack{k=1\\(k,\beta)\in S}}^{N} \sum_{\substack{r=1\\(r,\beta)\in S}}^{k-1} C_{ij\alpha}^{\mathrm{con}} C_{kr\beta}^{\mathrm{con}}\right] - \\
&\sum_{\alpha=1}^{\mathcal{L}} \sum_{\substack{i=1\\(i,\alpha)\in S}}^{N} \sum_{\substack{j=1\\(j,\alpha)\in S}}^{i-1} \sum_{\beta=1}^{\mathcal{L}} \sum_{\substack{k=1\\(k,\beta)\in S}}^{N} \sum_{\substack{r=1\\(r,\beta)\in S}}^{k-1} C_{ij\alpha} C_{kr\beta} \\
=&\sum_{\alpha=1}^{\mathcal{L}} \sum_{\substack{i=1\\(i,\alpha)\in S}}^{N} \sum_{\substack{j=1\\(j,\alpha)\in S}}^{i-1} \sum_{\beta=1}^{\mathcal{L}} \sum_{\substack{k=1\\(k,\beta)\in S}}^{N} \sum_{\substack{r=1\\(r,\beta)\in S}}^{k-1} \left(\mathrm{E}[C_{ij\alpha}^{\mathrm{con}} C_{kr\beta}^{\mathrm{con}}] - C_{ij\alpha} C_{kr\beta}\right) \\
=&\sum_{\alpha=1}^{\mathcal{L}} \sum_{\substack{i=1\\(i,\alpha)\in S}}^{N} \sum_{\substack{j=1\\(j,\alpha)\in S}}^{i-1} \sum_{\beta=1}^{\mathcal{L}} \sum_{\substack{k=1\\(k,\beta)\in S}}^{N} \sum_{\substack{r=1\\(r,\beta)\in S}}^{k-1} \left\{\mathrm{E}\left[\left(\frac{1}{L}\sum_{l=1}^{L} x_{il\alpha} x_{jl\alpha}\right)\left(\frac{1}{L}\sum_{l=1}^{L} x_{kl\beta} x_{rl\beta}\right)\right] - C_{ij\alpha} C_{kr\beta}\right\} \\
=&\sum_{\alpha=1}^{\mathcal{L}} \sum_{\substack{i=1\\(i,\alpha)\in S}}^{N} \sum_{\substack{j=1\\(j,\alpha)\in S}}^{i-1} \sum_{\beta=1}^{\mathcal{L}} \sum_{\substack{k=1\\(k,\beta)\in S}}^{N} \sum_{\substack{r=1\\(r,\beta)\in S}}^{k-1} \left(\frac{1}{L^2}\mathrm{E}\left[\sum_{l=1}^{L}\sum_{l'=1}^{L} x_{il\alpha} x_{jl\alpha} x_{kl'\beta} x_{rl'\beta}\right] - C_{ij\alpha} C_{kr\beta}\right),
\end{aligned}
\tag{S23}
$$

where L is the number of samples we draw from the N-variate multivariate normal distribution. Now, using the fact that different samples are independent, we obtain

$$\begin{aligned}
&\frac{1}{L^2}\mathrm{E}\left[\sum_{l=1}^{L}\sum_{l'=1}^{L} x_{il\alpha}x_{jl\alpha}x_{kl'\beta}x_{rl'\beta}\right] - C_{ij\alpha}C_{kr\beta} \\
=&\frac{1}{L^2}\sum_{l=1}^{L}\sum_{\substack{l'=1\\ l'\neq l}}^{L}\mathrm{E}[x_{il\alpha}x_{jl\alpha}x_{kl'\beta}x_{rl'\beta}] + \frac{1}{L^2}\sum_{l=1}^{L}\mathrm{E}[x_{il\alpha}x_{jl\alpha}x_{kl\beta}x_{rl\beta}] - C_{ij\alpha}C_{kr\beta} \\
=&\frac{1}{L^2}\sum_{l=1}^{L}\mathrm{E}[x_{il\alpha}x_{jl\alpha}]\cdot\sum_{\substack{l'=1\\ l'\neq l}}^{L}\mathrm{E}[x_{kl'\beta}x_{rl'\beta}] + \frac{1}{L^2}\sum_{l=1}^{L}\mathrm{E}[x_{il\alpha}x_{jl\alpha}x_{kl\beta}x_{rl\beta}] - C_{ij\alpha}C_{kr\beta} \\
=&\frac{1}{L^2}\cdot LC_{ij\alpha}\cdot(L-1)C_{kr\beta} + \frac{1}{L^2}\sum_{l=1}^{L}\mathrm{E}[x_{il\alpha}x_{jl\alpha}x_{kl\beta}x_{rl\beta}] - C_{ij\alpha}C_{kr\beta} \\
=&\frac{(L-1)}{L}C_{ij\alpha}C_{kr\beta} + \frac{1}{L^2}\sum_{l=1}^{L}\mathrm{E}[x_{il\alpha}x_{jl\alpha}x_{kl\beta}x_{rl\beta}] - C_{ij\alpha}C_{kr\beta} \\
=&-\frac{1}{L}C_{ij\alpha}C_{kr\beta} + \frac{1}{L^2}\sum_{l=1}^{L}\mathrm{E}[x_{il\alpha}x_{jl\alpha}x_{kl\beta}x_{rl\beta}]. \qquad \text{(S24)}
\end{aligned}$$

By substituting Eq. (S24) into Eq. (S23), using the fact that the covariance matrices C_α and C_β are independent when $\beta \neq \alpha$, and using Isserlis' Theorem [12], we obtain

$$
\begin{aligned}
\mathrm{Var}\left[\sum_{\alpha=1}^{\mathcal{L}} \sum_{\substack{i=1\\(i,\alpha)\in S}}^{N} \sum_{\substack{j=1\\(j,\alpha)\in S}}^{i-1} C_{ij\alpha}^{\mathrm{con}}\right] =& \sum_{\alpha=1}^{\mathcal{L}} \sum_{\substack{i=1\\(i,\alpha)\in S}}^{N} \sum_{\substack{j=1\\(j,\alpha)\in S}}^{i-1} \sum_{\beta=1}^{\mathcal{L}} \sum_{\substack{k=1\\(k,\beta)\in S}}^{N} \sum_{\substack{r=1\\(r,\beta)\in S}}^{k-1} \left(-\frac{1}{L} C_{ij\alpha} C_{kr\beta} + \frac{1}{L^2} \sum_{l=1}^{L} \mathrm{E}[x_{il\alpha} x_{jl\alpha} x_{kl\beta} x_{rl\beta}]\right) \\
=& \sum_{\alpha=1}^{\mathcal{L}} \sum_{\substack{i=1\\(i,\alpha)\in S}}^{N} \sum_{\substack{j=1\\(j,\alpha)\in S}}^{i-1} \sum_{\substack{\beta=1\\ \beta\neq\alpha}}^{\mathcal{L}} \sum_{\substack{k=1\\(k,\beta)\in S}}^{N} \sum_{\substack{r=1\\(r,\beta)\in S}}^{k-1} \left(-\frac{1}{L} C_{ij\alpha} C_{kr\beta} + \frac{1}{L^2} \sum_{l=1}^{L} \mathrm{E}[x_{il\alpha} x_{jl\alpha} x_{kl\beta} x_{rl\beta}]\right) \\
&+ \sum_{\alpha=1}^{\mathcal{L}} \sum_{\substack{i=1\\(i,\alpha)\in S}}^{N} \sum_{\substack{j=1\\(j,\alpha)\in S}}^{i-1} \sum_{\substack{k=1\\(k,\alpha)\in S}}^{N} \sum_{\substack{r=1\\(r,\alpha)\in S}}^{k-1} \left(-\frac{1}{L} C_{ij\alpha} C_{kr\alpha} + \frac{1}{L^2} \sum_{l=1}^{L} \mathrm{E}[x_{il\alpha} x_{jl\alpha} x_{kl\alpha} x_{rl\alpha}]\right) \\
=& -\frac{1}{L}\left(\sum_{\alpha=1}^{\mathcal{L}} \sum_{\substack{i=1\\(i,\alpha)\in S}}^{N} \sum_{\substack{j=1\\(j,\alpha)\in S}}^{i-1} C_{ij\alpha}\right)\left(\sum_{\substack{\beta=1\\ \beta\neq\alpha}}^{\mathcal{L}} \sum_{\substack{k=1\\(k,\beta)\in S}}^{N} \sum_{\substack{r=1\\(r,\beta)\in S}}^{k-1} C_{kr\beta}\right) \\
&+ \frac{1}{L^2} \sum_{l=1}^{L} \left(\sum_{\alpha=1}^{\mathcal{L}} \sum_{\substack{i=1\\(i,\alpha)\in S}}^{N} \sum_{\substack{j=1\\(j,\alpha)\in S}}^{i-1} \mathrm{E}[x_{il\alpha} x_{jl\alpha}]\right)\left(\sum_{\substack{\beta=1\\ \beta\neq\alpha}}^{\mathcal{L}} \sum_{\substack{k=1\\(k,\beta)\in S}}^{N} \sum_{\substack{r=1\\(r,\beta)\in S}}^{k-1} \mathrm{E}[x_{kl\beta} x_{rl\beta}]\right) \\
&- \frac{1}{L} \sum_{\alpha=1}^{\mathcal{L}} \left(\sum_{\substack{i=1\\(i,\alpha)\in S}}^{N} \sum_{\substack{j=1\\(j,\alpha)\in S}}^{i-1} C_{ij\alpha}\right)\left(\sum_{\substack{k=1\\(k,\alpha)\in S}}^{N} \sum_{\substack{r=1\\(r,\alpha)\in S}}^{k-1} C_{kr\alpha}\right) \\
&+ \frac{1}{L^2} \sum_{l=1}^{L} \left\{\sum_{\alpha=1}^{\mathcal{L}} \sum_{\substack{i=1\\(i,\alpha)\in S}}^{N} \sum_{\substack{j=1\\(j,\alpha)\in S}}^{i-1} \sum_{\substack{k=1\\(k,\alpha)\in S}}^{N} \sum_{\substack{r=1\\(r,\alpha)\in S}}^{k-1} (\mathrm{E}[x_{il\alpha} x_{jl\alpha}] \cdot \mathrm{E}[x_{kl\alpha} x_{rl\alpha}] \right. \\
&\left. + \mathrm{E}[x_{il\alpha} x_{kl\alpha}] \cdot \mathrm{E}[x_{jl\alpha} x_{rl\alpha}] + \mathrm{E}[x_{il\alpha} x_{rl\alpha}] \cdot \mathrm{E}[x_{jl\alpha} x_{kl\alpha}])\right\} \\
=& -\frac{1}{L}\left(\sum_{\alpha=1}^{\mathcal{L}} \sum_{\substack{i=1\\(i,\alpha)\in S}}^{N} \sum_{\substack{j=1\\(j,\alpha)\in S}}^{i-1} C_{ij\alpha}\right)\left(\sum_{\substack{\beta=1\\ \beta\neq\alpha}}^{\mathcal{L}} \sum_{\substack{k=1\\(k,\beta)\in S}}^{N} \sum_{\substack{r=1\\(r,\beta)\in S}}^{k-1} C_{kr\beta}\right) \\
&+ \frac{1}{L^2} \cdot L \left(\sum_{\alpha=1}^{\mathcal{L}} \sum_{\substack{i=1\\(i,\alpha)\in S}}^{N} \sum_{\substack{j=1\\(j,\alpha)\in S}}^{i-1} C_{ij\alpha}\right)\left(\sum_{\substack{\beta=1\\ \beta\neq\alpha}}^{\mathcal{L}} \sum_{\substack{k=1\\(k,\beta)\in S}}^{N} \sum_{\substack{r=1\\(r,\beta)\in S}}^{k-1} C_{kr\beta}\right) \\
&- \frac{1}{L} \sum_{\alpha=1}^{\mathcal{L}} \left(\sum_{\substack{i=1\\(i,\alpha)\in S}}^{N} \sum_{\substack{j=1\\(j,\alpha)\in S}}^{i-1} C_{ij\alpha}\right)\left(\sum_{\substack{k=1\\(k,\alpha)\in S}}^{N} \sum_{\substack{r=1\\(r,\alpha)\in S}}^{k-1} C_{kr\alpha}\right) \\
&+ \frac{1}{L^2} \cdot L \left[\sum_{\alpha=1}^{\mathcal{L}} \sum_{\substack{i=1\\(i,\alpha)\in S}}^{N} \sum_{\substack{j=1\\(j,\alpha)\in S}}^{i-1} \sum_{\substack{k=1\\(k,\alpha)\in S}}^{N} \sum_{\substack{r=1\\(r,\alpha)\in S}}^{k-1} (C_{ij\alpha} C_{kr\alpha} + C_{ik\alpha} C_{jr\alpha} + C_{ir\alpha} C_{jk\alpha})\right].
\end{aligned}
\tag{S25}
$$

By combining the first and third terms in Eq. (S25), we obtain

$$
\begin{aligned}
\mathrm{Var}\left[\sum_{\alpha=1}^{\mathcal{L}}\sum_{\substack{i=1\\(i,\alpha)\in S}}^{N}\sum_{\substack{j=1\\(j,\alpha)\in S}}^{i-1} C_{ij\alpha}^{\mathrm{con}}\right] =& -\frac{1}{L}\left(\sum_{\alpha=1}^{\mathcal{L}}\sum_{\substack{i=1\\(i,\alpha)\in S}}^{N}\sum_{\substack{j=1\\(j,\alpha)\in S}}^{i-1} C_{ij\alpha}\right)\left(\sum_{\beta=1}^{\mathcal{L}}\sum_{\substack{k=1\\(k,\beta)\in S}}^{N}\sum_{\substack{r=1\\(r,\beta)\in S}}^{k-1} C_{kr\beta}\right)\\
&+\frac{1}{L}\left(\sum_{\alpha=1}^{\mathcal{L}}\sum_{\substack{i=1\\(i,\alpha)\in S}}^{N}\sum_{\substack{j=1\\(j,\alpha)\in S}}^{i-1} C_{ij\alpha}\right)\left(\sum_{\substack{\beta=1\\ \beta\neq\alpha}}^{\mathcal{L}}\sum_{\substack{k=1\\(k,\beta)\in S}}^{N}\sum_{\substack{r=1\\(r,\beta)\in S}}^{k-1} C_{kr\beta}\right)\\
&+\frac{1}{L}\left[\sum_{\alpha=1}^{\mathcal{L}}\sum_{\substack{i=1\\(i,\alpha)\in S}}^{N}\sum_{\substack{j=1\\(j,\alpha)\in S}}^{i-1}\sum_{\substack{k=1\\(k,\alpha)\in S}}^{N}\sum_{\substack{r=1\\(r,\alpha)\in S}}^{k-1}\left(C_{ij\alpha}C_{kr\alpha}+C_{ik\alpha}C_{jr\alpha}+C_{ir\alpha}C_{jk\alpha}\right)\right]\\
=& -\frac{1}{L}\left(\sum_{\alpha=1}^{\mathcal{L}}\sum_{\substack{i=1\\(i,\alpha)\in S}}^{N}\sum_{\substack{j=1\\(j,\alpha)\in S}}^{i-1} C_{ij\alpha}\right)^2\\
&+\frac{1}{L}\left(\sum_{\alpha=1}^{\mathcal{L}}\sum_{\substack{i=1\\(i,\alpha)\in S}}^{N}\sum_{\substack{j=1\\(j,\alpha)\in S}}^{i-1} C_{ij\alpha}\right)\left(\sum_{\substack{\beta=1\\ \beta\neq\alpha}}^{\mathcal{L}}\sum_{\substack{k=1\\(k,\beta)\in S}}^{N}\sum_{\substack{r=1\\(r,\beta)\in S}}^{k-1} C_{kr\beta}\right)\\
&+\frac{1}{L}\left(\sum_{\alpha=1}^{\mathcal{L}}\sum_{\substack{i=1\\(i,\alpha)\in S}}^{N}\sum_{\substack{j=1\\(j,\alpha)\in S}}^{i-1}\sum_{\substack{k=1\\(k,\alpha)\in S}}^{N}\sum_{\substack{r=1\\(r,\alpha)\in S}}^{k-1} C_{ij\alpha}C_{kr\alpha}\right)\\
&+\frac{1}{L}\left[\sum_{\alpha=1}^{\mathcal{L}}\sum_{\substack{i=1\\(i,\alpha)\in S}}^{N}\sum_{\substack{j=1\\(j,\alpha)\in S}}^{i-1}\sum_{\substack{k=1\\(k,\alpha)\in S}}^{N}\sum_{\substack{r=1\\(r,\alpha)\in S}}^{k-1}\left(C_{ik\alpha}C_{jr\alpha}+C_{ir\alpha}C_{jk\alpha}\right)\right]\\
=& -\frac{1}{L}\left(\sum_{\alpha=1}^{\mathcal{L}}\sum_{\substack{i=1\\(i,\alpha)\in S}}^{N}\sum_{\substack{j=1\\(j,\alpha)\in S}}^{i-1} C_{ij\alpha}\right)^2+\frac{1}{L}\left(\sum_{\alpha=1}^{\mathcal{L}}\sum_{\substack{i=1\\(i,\alpha)\in S}}^{N}\sum_{\substack{j=1\\(j,\alpha)\in S}}^{i-1} C_{ij\alpha}\right)^2\\
&+\frac{1}{L}\left[\sum_{\alpha=1}^{\mathcal{L}}\sum_{\substack{i=1\\(i,\alpha)\in S}}^{N}\sum_{\substack{j=1\\(j,\alpha)\in S}}^{i-1}\sum_{\substack{k=1\\(k,\alpha)\in S}}^{N}\sum_{\substack{r=1\\(r,\alpha)\in S}}^{k-1}\left(C_{ik\alpha}C_{jr\alpha}+C_{ir\alpha}C_{jk\alpha}\right)\right]\\
=&\frac{1}{L}\left[\sum_{\alpha=1}^{\mathcal{L}}\sum_{\substack{i=1\\(i,\alpha)\in S}}^{N}\sum_{\substack{j=1\\(j,\alpha)\in S}}^{i-1}\sum_{\substack{k=1\\(k,\alpha)\in S}}^{N}\sum_{\substack{r=1\\(r,\alpha)\in S}}^{k-1}\left(C_{ik\alpha}C_{jr\alpha}+C_{ir\alpha}C_{jk\alpha}\right)\right]. \qquad \text{(S26)}
\end{aligned}
$$

S3 Z scores for the average distance between pairs of genes on each chromosome separately in each community

We show in Table S3 the Z scores for the average distance between pairs of genes on each chromosome and each community with $\gamma = 1$. We show the corresponding results with $\gamma = 3$ in Table S4. We only calculated the Z scores for the chromosome-community pairs with at least 3 genes. In these tables, N/A implies that either there are less than 3 genes, or the standard deviation of the average distance is equal to 0 because

Table S3: Z scores for the average distance between pairs of genes on each chromosome and each significant community detected with $\gamma = 1$. Comm. denotes community and Chr denotes chromosome.

$\gamma = 1$					
	Comm.				
Chr	1	2	3	4	5
1	−2.093	−1.526	−0.122	−1.466	1.272
2	N/A	−0.767	0.061	2.129	−0.725
3	N/A	N/A	0.113	1.101	N/A
4	N/A	N/A	N/A	−0.110	−2.573
5	N/A	N/A	N/A	N/A	N/A
6	0.606	N/A	N/A	0.564	−0.065
7	N/A	−1.938	−1.842	0.968	−0.302
8	N/A	N/A	N/A	N/A	N/A
9	N/A	N/A	N/A	−0.722	N/A
10	N/A	−2.336	0.413	N/A	N/A
11	N/A	N/A	−2.156	0.077	0.454
12	N/A	N/A	−1.376	−0.905	−0.260
13	N/A	N/A	N/A	N/A	N/A
14	N/A	N/A	0.438	N/A	0.180
15	N/A	N/A	N/A	N/A	N/A
16	−0.091	0.737	0.100	N/A	N/A
17	1.535	N/A	−2.160	−0.579	−0.787
18	N/A	N/A	N/A	N/A	N/A
19	0.235	N/A	−0.521	1.592	1.894
20	N/A	N/A	N/A	N/A	0.791
21	N/A	N/A	N/A	N/A	N/A
22	N/A	N/A	N/A	N/A	−1.780
X	N/A	N/A	N/A	N/A	N/A
Y	N/A	N/A	N/A	N/A	N/A
M	N/A	N/A	N/A	N/A	−2.061

every random selection of genes is the same gene set. The latter event occurs when all the genes on that chromosome are associated with the same community.

S4 Gene set enrichment analysis results

We show the two most significant GO:BP and HP results from g:Profiler for the top 50 highly expressed genes out of the 203 genes in the network in each tissue in Table S5. See [13] for the entire output from g:Profiler, i.e., the list of all significant GO:BP and HP results, for the top 50 genes in pancreas; see [14] for the top 50 genes in salivary gland; see [15] for the top 50 genes in mammary gland; and see [16] for the top 50 genes in skin.

We show the two most significant GO:BP and HP results from g:Profiler for each community in the partition of the multilayer correlation matrix with $\gamma = 3$ in Table S6. See [17] for the entire output from g:Profiler, i.e., the list of all significant GO:BP and HP results, for the genes in community 1; see [18] for community 2; see [19] for community 3; see [20] for community 4; see [21] for community 5; see [22] for community 6, and see [23] for community 7.

Table S4: Z scores for the average distance between pairs of genes on each chromosome and each significant community detected with $\gamma = 3$. Comm. denotes community and Chr denotes chromosome.

$\gamma = 3$							
	Comm.						
Chr	1	2	3	4	5	6	7
1	−2.808	0.116	−0.447	N/A	−0.482	N/A	N/A
2	−0.671	N/A	N/A	N/A	2.094	−2.220	N/A
3	N/A	N/A	N/A	N/A	N/A	N/A	N/A
4	N/A	N/A	N/A	N/A	−1.993	N/A	N/A
5	N/A	N/A	N/A	N/A	N/A	N/A	N/A
6	N/A	0.573	N/A	N/A	N/A	N/A	N/A
7	−1.643	N/A	N/A	N/A	−2.616	N/A	N/A
8	N/A	N/A	N/A	N/A	N/A	N/A	N/A
9	N/A	N/A	N/A	N/A	N/A	N/A	N/A
10	N/A	N/A	N/A	N/A	0.293	N/A	N/A
11	N/A	N/A	N/A	N/A	−2.902	N/A	N/A
12	N/A	1.729	N/A	N/A	−0.577	N/A	N/A
13	N/A	N/A	N/A	N/A	N/A	N/A	N/A
14	N/A	N/A	N/A	N/A	N/A	−1.756	N/A
15	N/A	N/A	N/A	N/A	N/A	N/A	N/A
16	−0.416	0.920	N/A	N/A	1.016	N/A	N/A
17	N/A	1.639	N/A	N/A	−2.175	N/A	N/A
18	N/A	N/A	N/A	N/A	N/A	N/A	N/A
19	N/A	−1.142	N/A	N/A	−0.179	N/A	N/A
20	N/A	N/A	N/A	N/A	N/A	N/A	N/A
21	N/A	N/A	N/A	N/A	N/A	N/A	N/A
22	N/A	N/A	N/A	N/A	N/A	N/A	N/A
X	N/A	N/A	N/A	N/A	N/A	N/A	N/A
Y	N/A	N/A	N/A	N/A	N/A	N/A	N/A
M	N/A	N/A	N/A	N/A	N/A	N/A	N/A

Table S5: Results of the gene set enrichment analysis for the top 50 highly expressed genes out of the 203 genes in the network in each tissue.

Tissue	Top significant terms from GO:BP	p value	Top significant terms from HP	p value
pancreas	oxidative phosphorylation	$6.39 \cdot 10^{-15}$	recurrent pancreatitis	$1.44 \cdot 10^{-22}$
	aerobic electron transport chain	$4.69 \cdot 10^{-14}$	mitochondrial inheritance	$1.79 \cdot 10^{-20}$
salivary gland	oxidative phosphorylation	$7.02 \cdot 10^{-16}$	mitochondrial inheritance	$6.33 \cdot 10^{-21}$
	aerobic electron transport chain	$7.37 \cdot 10^{-15}$	centrocecal scotoma	$3.23 \cdot 10^{-20}$
mammary gland	oxidative phosphorylation	$6.35 \cdot 10^{-15}$	mitochondrial inheritance	$9.67 \cdot 10^{-20}$
	mitochondrial ATP synthesis coupled electron transport	$4.77 \cdot 10^{-14}$	centrocecal scotoma	$3.63 \cdot 10^{-19}$
skin	oxidative phosphorylation	$7.28 \cdot 10^{-15}$	mitochondrial inheritance	$5.06 \cdot 10^{-19}$
	aerobic electron transport chain	$5.35 \cdot 10^{-14}$	centrocecal scotoma	$1.59 \cdot 10^{-18}$

Table S6: Results of the gene set enrichment analysis for the communities of the multilayer correlation matrix with $\gamma = 3$. Comm. denotes community.

Comm.	Top significant terms from GO:BP	p value	Top significant terms from HP	p value
1	oxidative phosphorylation	$5.90 \cdot 10^{-11}$	recurrent pancreatitis	$1.78 \cdot 10^{-19}$
	aerobic electron transport chain	$1.05 \cdot 10^{-10}$	mitochondrial inheritance	$1.06 \cdot 10^{-17}$
2	keratinocyte differentiation	$8.26 \cdot 10^{-25}$	palmoplantar keratoderma	$3.05 \cdot 10^{-7}$
	epidermis development	$1.90 \cdot 10^{-24}$	hyperkeratosis	$3.05 \cdot 10^{-7}$
3	retina homeostasis	$2.52 \cdot 10^{-10}$	leber optic atophy	$6.65 \cdot 10^{-15}$
	oxidative phosphorylation	$2.23 \cdot 10^{-9}$	mitochondrial inheritance	$6.65 \cdot 10^{-15}$
4	skin development	$3.23 \cdot 10^{-13}$	alopecia	$8.30 \cdot 10^{-5}$
	intermediate filament organization	$2.64 \cdot 10^{-12}$	nail dystrophy	$8.30 \cdot 10^{-5}$
5	keratinization	$1.95 \cdot 10^{-19}$	palmoplantar blistering	$1.71 \cdot 10^{-6}$
	epidermis development	$1.95 \cdot 10^{-19}$	palmoplantar keratoderma	$2.02 \cdot 10^{-5}$
6	positive regulation of respiratory burst	$5.36 \cdot 10^{-8}$	N/A	N/A
	regulation of respiratory burst	$2.60 \cdot 10^{-7}$	N/A	N/A
7	adaptive thermogenesis	$1.73 \cdot 10^{-2}$	N/A	N/A
	fatty acid biosynthesis process	$1.73 \cdot 10^{-2}$	N/A	N/A

Supplementary References

[1] J Park and M E J Newman. Origin of degree correlations in the internet and other networks. *Physical Review E*, 68(2):026112, 2003.

[2] M Catanzaro, M Boguñá, and R Pastor-Satorras. Generation of uncorrelated random scale-free networks. *Physical Review E*, 71(2):027103, 2005.

[3] S Maslov, K Sneppen, and A Zaliznyak. Detection of topological patterns in complex networks: correlation profile of the internet. *Physica A*, 333:529–540, 2004.

[4] D Garlaschelli and M I Loffredo. Generalized bose-fermi statistics and structural correlations in weighted networks. *Physical Review Letters*, 102(3):038701, 2009.

[5] J Park and M E J Newman. Statistical mechanics of networks. *Physical Review E*, 70(6):066117, 2004.

[6] G Cimini, T Squartini, F Saracco, D Garlaschelli, A Gabrielli, and G Caldarelli. The statistical physics of real-world networks. *Nature Reviews Physics*, 1(1):58–71, 2019.

[7] N Vallarano, M Bruno, E Marchese, G Trapani, F Saracco, G Cimini, M Zanon, and T Squartini. Fast and scalable likelihood maximization for Exponential Random Graph Models with local constraints. *Scientific Reports*, 11:15227, 2021.

[8] F Radicchi, C Castellano, F Cecconi, V Loreto, and D Parisi. Defining and identifying communities in networks. *Proceedings of the National Academy of Sciences of the United States of America*, 101(9):2658–2663, 2004.

[9] J Yang and J Leskovec. Defining and evaluating network communities based on ground-truth. *Knowledge and Information Systems*, 42(1):181–213, 2015.

[10] Y H Wang. On the number of successes in independent trials. *Statistica Sinica*, 3(2):295–312, 1993.

[11] J Shi and J Malik. Normalized cuts and image segmentation. *IEEE Transactions on Pattern Analysis and Machine Intelligence*, 22(8):888–905, 2000.

[12] L Isserlis. On a formula for the product-moment coefficient of any order of a normal frequency distribution in any number of variables. *Biometrika*, 12(1/2):134–139, 1918.

[13] g:Profiler g:GOSt (pancreas). `https://biit.cs.ut.ee/gplink/l/QbFURpSESM`. Accessed: 27 April 2023.

[14] g:Profiler g:GOSt (salivary gland). `https://biit.cs.ut.ee/gplink/l/ajM--6FRTY`. Accessed: 27 April 2023.

[15] g:Profiler g:GOSt (mammary gland). `https://biit.cs.ut.ee/gplink/l/aDH27OkWTD`. Accessed: 27 April 2023.

[16] g:Profiler g:GOSt (skin). `https://biit.cs.ut.ee/gplink/l/F2kZch7qRG`. Accessed: 27 April 2023.

[17] g:Profiler g:GOSt (community 1). `https://biit.cs.ut.ee/gplink/l/TLbKa39zRU`. Accessed: 27 April 2023.

[18] g:Profiler g:GOSt (community 2). `https://biit.cs.ut.ee/gplink/l/OueSgf4HRJ`. Accessed: 27 April 2023.

[19] g:Profiler g:GOSt (community 3). `https://biit.cs.ut.ee/gplink/l/T21OqL34SQ`. Accessed: 27 April 2023.

[20] g:Profiler g:GOSt (community 4). `https://biit.cs.ut.ee/gplink/l/6p4rYKFtQE`. Accessed: 27 April 2023.

[21] g:Profiler g:GOSt (community 5). https://biit.cs.ut.ee/gplink/l/JGyRZKeZQf. Accessed: 27 April 2023.

[22] g:Profiler g:GOSt (community 6). https://biit.cs.ut.ee/gplink/l/-xMfgdJbSo. Accessed: 27 April 2023.

[23] g:Profiler g:GOSt (community 7). https://biit.cs.ut.ee/gplink/l/eCuRxFu4QT. Accessed: 27 April 2023.
